# Effectiveness and safety of cefazolin vs antistaphylococcal penicillins for methicillin-susceptible *Staphylococcus aureus* bacteremia: a multicenter real-world evaluation using the international TriNetX database

**DOI:** 10.1128/aac.01765-25

**Published:** 2026-09-11

**Authors:** Natesan Ramsamy, Maïa Simon, Isabelle Adam, Gema Hernandez, Sarah Williford, Olivier Denquin, Céline Pulcini, Nathalie Thilly, Benjamin Lefèvre

**Affiliations:** 1 CHRU-Nancy, Service des Maladies Infectieuses et Tropicales, Université de Lorraine 137665, Nancy, France; 2 Département Méthodologie Promotion Investigation, Université de Lorraine, CHRU-Nancy 137665, Nancy, France; 3 Université de Lorraine, INSERM, INSPIIRE, Nancy, France; 4 TriNetX LLC 702875 , Cambridge, Massachusetts, USA; 5 CHRU-Nancy, Centre Régional en Antibiothérapie du Grand Est AntibioEst, Université de Lorraine 137665, Nancy, France; The Peter Doherty Institute for Infection and Immunity, Melbourne, Victoria, Australia

**Keywords:** methicillin-susceptible *Staphylococcus aureus*, bacteremia, antistaphylococcal penicillins, cefazolin, real-word evaluation, TriNetX

## Abstract

This study aimed to evaluate the effectiveness and safety of cefazolin vs antistaphylococcal penicillins (ASPs) in the treatment of methicillin-susceptible *Staphylococcus aureus* (MSSA) bacteremia. This phase IV, retrospective, comparative-effectiveness, multicenter study used data extracted from the international TriNetX analytics platform. Eligible patients developed monomicrobial MSSA bacteremia during hospitalization between 1 January 2015 and 31 December 2025 and received treatment with either cefazolin or ASPs. The primary outcome was all-cause mortality within 90 days. Safety outcomes included *Clostridioides difficile* infection within 90 days and renal, liver, allergic, and hematological disorders within 30 days. Survival analyses were conducted after propensity score matching. Among 110,275 patients with MSSA bacteremia identified, 3,908 (3.5%) met the inclusion criteria. After propensity score matching, 1,954 patients were included in each group. Treatment with cefazolin was associated with significantly reduced all-cause mortality within 90 days (9.7% vs 14.7%, HR = 0.620 [0.515–0.748]). Among the safety outcomes, *Clostridioides difficile* infection occurred less frequently in the cefazolin group (4.9% vs 7.20%, HR = 0.631 [0.482–0.827]). This real-world evaluation using the international TriNetX database suggests that, compared with ASPs, cefazolin is more effective and safer for the management of MSSA bacteremia.

**CLINICAL TRIALS:**

This study is registered with ClinicalTrials.gov as NCT07186894.

## INTRODUCTION

*Staphylococcus aureus* (SA) is a common cause of bacteremia and is associated with high mortality rates (1). SA bacteremia incidence ranges from 9 to 65 cases per 100,000 person-years, depending on the geographical region (2). Several studies have reported a rising incidence of SA bacteremia in recent decades, concomitant with a decline in the prevalence of methicillin-resistant strains (3, 4). In a recent meta-analysis by Bai et al. (5), all-cause mortality associated with SA bacteremia remained substantial: 18.1% at 1 month, 27.0% at 3 months, and 30.2% at 1 year. Factors influencing the prognosis of SA bacteremia have been identified in several publications (6). These include host-related factors (e.g., age, sex, and comorbidities), host‒pathogen interactions (e.g., source and setting of bacteremia and severity of the infection), pathogen-related factors (e.g., resistance and toxins), and patient management (e.g., antibiotic therapy and surgery) (6).

In the absence of specific international guidelines, the management of methicillin-susceptible *Staphylococcus aureus* (MSSA) bloodstream infections has primarily been based on expert opinion (7, 8). This may have contributed to considerable heterogeneity in clinical practices worldwide (9, 10), further compounded by the ongoing debate regarding the optimal first-line antibiotic choice (7, 11). For several decades, antistaphylococcal penicillins (ASPs), such as cloxacillin, oxacillin, and nafcillin, have been traditionally suggested as first-line agents, and cefazolin is considered a second-line therapy.

This opinion was largely based on studies suggesting that, compared with ASPs, cefazolin might be more susceptible to the inoculum effect (12–18). Consequently, international societies recommended ASPs as the first-line therapy for high-inoculum MSSA infections, such as endocarditis (19, 20). By extension, experts have also proposed ASPs as first-line treatments for MSSA bacteremia, irrespective of the presumed inoculum size (21–24). More recently, international guidelines have evolved, now placing cefazolin and ASPs as equivalent options for the treatment of high-inoculum MSSA infections (25, 26). This shift is supported by recent studies highlighting both the challenges in assessing the clinical relevance of the inoculum effect (27–31) and its occurrence with both cefazolin and ASPs (32, 33).

ASPs are known to be associated with higher rates of adverse events than cefazolin (34). Moreover, recurrent global shortages of penicillins have forced clinicians to use cefazolin as a first-line treatment (35). In this context, several meta-analyses based on observational studies and two randomized controlled trials (RCTs) have suggested that, compared with ASPs, cefazolin has comparable or better effectiveness on all-cause mortality, along with a more favorable safety profile. These findings are summarized in Table S1 (34, 36–44). Nonetheless, large real-world evaluations are necessary to complement clinical trials, which typically include highly selected and homogenous populations. In this context, the TriNetX platform provides access to real-world, real-time global hospital health data, enabling comparative analyses in an international setting.

This study aimed to evaluate the effectiveness and safety of cefazolin vs ASPs in the treatment of MSSA bacteremia using the TriNetX database.

## MATERIALS AND METHODS

### Study design

This study was a phase IV, retrospective, comparative-effectiveness, multicenter international study conducted on real-world data from patients hospitalized for MSSA bacteremia between 2015 and 2025 in healthcare organizations (HCOs) participating in the TriNetX network.

### Participants

Patients were eligible if they were hospitalized between 1 January 2015 and 31 December 2025, with monomicrobial bacteremia caused by MSSA, or if they developed MSSA bacteremia during hospitalization for another reason. The cefazolin group included patients who received at least one injectable prescription of cefazolin within the 2 days preceding or the 3 days following the diagnosis of MSSA bacteremia. The ASP group included patients who received at least one injectable prescription of an ASP, including flucloxacillin, floxacillin, cloxacillin, oxacillin, nafcillin, or dicloxacillin, within the same time frame.

The exclusion criteria included (i) the presence of any other documented bacterial infection or (ii) receipt of both cefazolin and any ASP within the 3 months preceding or following the index event. These exclusions were applied to minimize the potential impact of such factors on effectiveness and safety outcomes.

For each patient, the index event date was defined as the first day of cefazolin or ASP treatment for MSSA bacteremia (day 0). This date served as the inclusion date; however, the follow-up period commenced on the following day (day 1), as the TriNetX platform does not allow outcomes occurring on day 0 to be distinguished as occurring before or after the index event.

In cases where a patient experienced multiple episodes of MSSA bacteremia during the study period, only the first documented episode of MSSA bacteremia was considered.

Detailed criteria for group definitions are provided in Tables S2 and S3.

### Data source and platform

This retrospective cohort study used the TriNetX Global federated network, a research platform that enables privacy-protective analyses of routinely collected electronic health record data contributed by participating healthcare organizations under site-specific governance and contractual collaboration (45). Data remain within each participating organization’s environment (or an organization-controlled hosted appliance) and are queried through a federated architecture; only aggregated, de-identified results are returned to the analytic environment rather than patient-level records. Contributed data are harmonized by mapping to controlled clinical terminologies and transformation into a common data schema and are subject to structured data quality assessment (including conformance, completeness, and plausibility checks) and cleaning during ingestion and refresh, including rejection of records that do not meet quality standards. To protect privacy in aggregate reporting, the platform applies small-cell suppression and obfuscation rules for patient counts and certain outputs. More information on the database is available online (46).

### Data considered

Data available within the TriNetX database are categorized into demographics, diagnoses, genomics, laboratory results, medications, and procedures. No additional data were available. In particular, information on the type or geographical location of contributing HCOs, the dosage and duration of antibiotic therapy, and the causes of death was not available.

Regardless of the covariate studied, only those documented in the patient’s medical record and coded at the time of hospital discharge or during any subsequent hospital follow-up could be included. Consequently, the quality and completeness of the data depended on the accuracy and timeliness of this documentation. Post-discharge data were accessible solely if recorded through continued follow-up within the same hospital.

Several covariates were used to characterize the population and to perform propensity score matching: (i) sociodemographic characteristics (age and sex); (ii) Charlson comorbidity index (CCI)-related comorbidities (47); (iii) variables associated with an increased risk of complicated infection (e.g., valvular diseases and indwelling devices); and (iv) variables available within the TriNetX database that are commonly described as prognostic factors of *Staphylococcus aureus* bacteremia (6).

As the original CCI variables were not directly available within TriNetX, CCI-related comorbidities were approximated using the closest corresponding variables available in the database. Likewise, among the prognostic factors previously reported for *Staphylococcus aureus* bacteremia, only those available within TriNetX could be included in the analyses. Most of these variables corresponded to host-related factors, including age, sex, and underlying comorbidities. For all covariates included in the propensity score matching procedure, the presence of the condition was assessed using all available patient records from birth up to the index event. Definitions of each covariate used for this study are provided in Table S4.

To characterize the population after the index event in the propensity score-matched cohorts, covariates related to documented infectious foci (e.g., central nervous system infections, endocarditis, and osteomyelitis) and co-prescribed antibiotics (e.g., quinolones, trimethoprim, and macrolides) were considered within the 30-day period following the index event. Definitions of each covariate used for this study are provided in Tables S5 and S6.

### Outcomes

The primary outcome for assessing the therapeutic effectiveness of cefazolin compared with ASPs was all-cause mortality within 90 days. Secondary effectiveness outcomes included all-cause mortality within 7 days, 30 days, and 1 year.

To evaluate the robustness of our results, a sensitivity analysis was conducted focusing on the primary outcome (i.e., 90-day all-cause mortality). This complementary analysis examined a subgroup of patients with high-inoculum infections, which are associated with higher mortality in the literature, namely, MSSA native or device-related endocarditis and central nervous system infections (6). The criteria used to define these subgroups are presented in Tables S7 and S8.

Safety was evaluated through the following secondary outcomes: *Clostridioides difficile* infection within 90 days; renal impairment; and liver dysfunction, allergic reactions, and hematological disorder, which may or may not be related to the antibiotic treatment, all of which were assessed on day 30. A 30-day follow-up period was selected for most safety outcomes, as this time frame was considered the most appropriate for observing these outcomes, particularly in the absence of data on antibiotic treatment duration. For *C. difficile* infection, a 90-day window was chosen to allow sufficient time for the detection of this specific complication. *Clostridioides difficile* infection was defined by either a recorded clinical diagnosis or a procedure or laboratory test consistent with its diagnosis. Renal impairment was defined as a clinician-recorded diagnosis of acute kidney injury, as the standardized laboratory data required to confirm this outcome according to consensus criteria were not available in the TriNetX database. Liver dysfunction was defined as any clinical diagnoses directly or indirectly indicative of hepatic impairment, including hepatic cytolysis and cholestasis. Allergic reactions were defined as any allergy-related diagnoses recorded in the TriNetX database, including anaphylaxis and drug-induced cutaneous reactions. Hematological disorders included any diagnoses of hematological abnormalities documented during follow-up, irrespective of the cell lineage involved. Definitions of these outcomes are provided in Table S9.

### Statistical analysis

Categorical variables are presented as numbers and percentages, whereas continuous variables are presented as the means and standard deviations.

To ensure comparability between the cefazolin and ASP cohorts, 1:1 propensity score matching was performed, including patient characteristics occurring prior to or at the date of the index event that were (i) significantly different between cohorts in the bivariate analyses and/or (ii) clinically relevant and known to confound mortality outcomes at the index event date (6). Greedy nearest-neighbor matching was used, with a caliper of 0.1 pooled standard deviations.

Patient characteristics occurring before or at the date of the index event were compared between groups (cefazolin vs ASPs cohorts) both before and after propensity score matching by calculating absolute standardized differences (StDiffs). In addition, the following post-index event characteristics were also described (but not included in the propensity score calculation) as they could influence the outcomes: (i) specific infectious foci associated with bacteremia (e.g., vertebral osteomyelitis and endocarditis) identified after the date of the index event and (ii) use of concomitant antibiotics alongside cefazolin or ASPs. Their definitions are provided in Tables S5 and S6.

Outcome analyses in the matched cohort included (i) survival analysis using Kaplan–Meier curves with comparisons by the log-rank test and (ii) Cox proportional hazards regression to estimate hazard ratios with 95% confidence intervals.

All patients who experienced an outcome before or on the day of the index event were excluded. Missing data were considered unavailable or absent for all types of variables and were not included in the analysis. Furthermore, the TriNetX platform does not support data imputation; consequently, no imputation procedures were performed to account for missing values.

All statistical analyses were performed using TriNetX platform software. The software used to generate these analyses included Java 11.0.16 (with Apache Commons Math 3.6.1), R 4.0.2 (with Hmisc1-1 and Survival 3.2-3), and Python 3.7 (utilizing lifelines 0.22.4, matplotlib 3.5.1, numpy 1.21.5, pandas 1.3.5, scipy 1.7.3, and statsmodels 0.13.2). A StDiff of <0.10 was considered to indicate a negligible difference in the mean or percentage of covariates between groups, and a two-tailed *P* value of <0.05 was considered statistically significant.

The methodology and statistical analyses were validated by clinicians, methodologists, and statisticians from Nancy Hospital and the TriNetX organization, and were registered on the ClinicalTrials platform prior to project initiation (NCT07186894).

## RESULTS

### Participants

**Fig 1:**
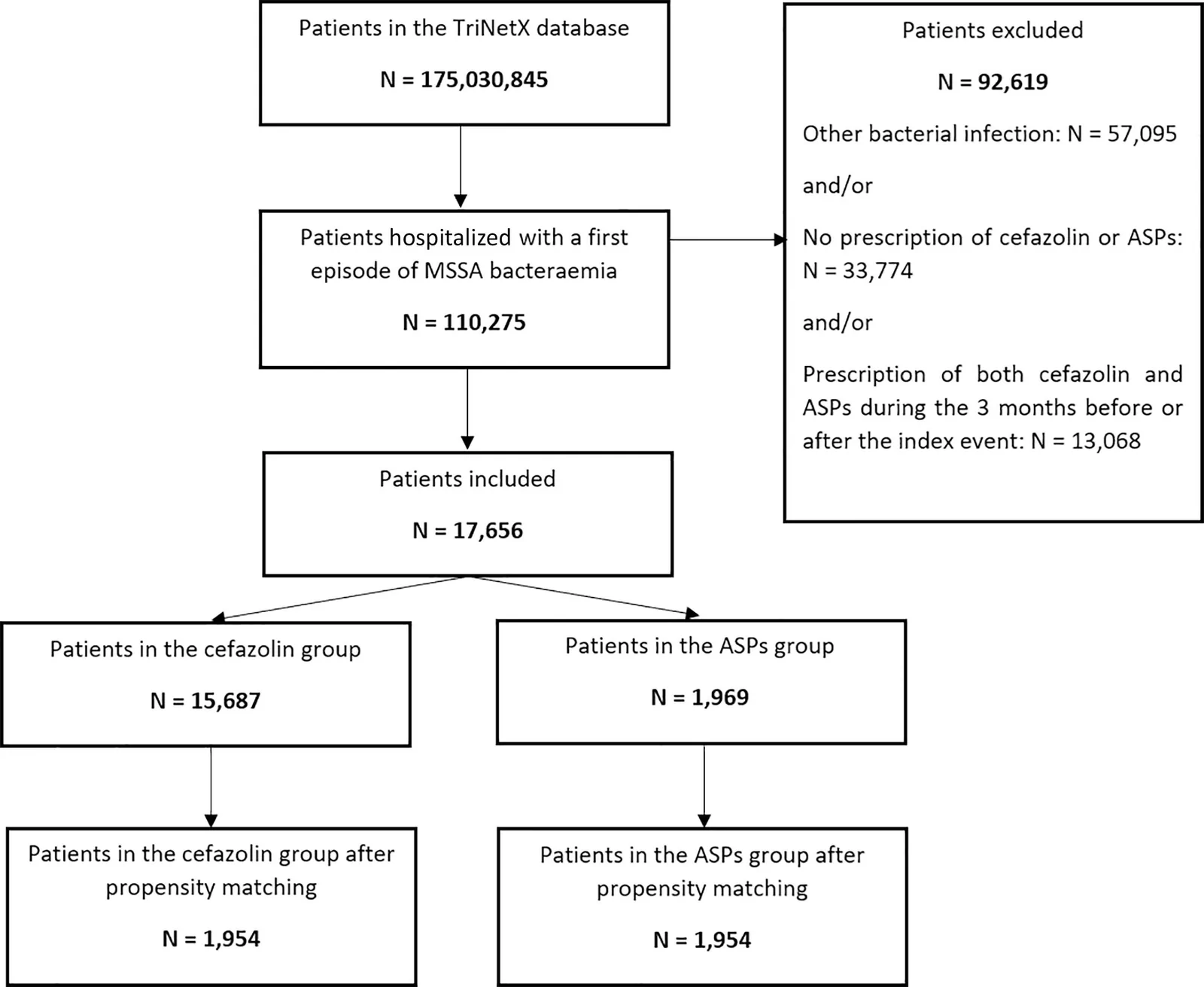
Flowchart illustrating the patient selection process.

Among the 110,275 patients hospitalized and diagnosed with MSSA bacteremia, 92,619 met the exclusion criteria (Fig. 1). Before propensity score matching, the cefazolin group included 15,687 patients, whereas the ASP group included 1,969 patients. After matching, each group included 1,954 patients.

In accordance with the anonymization policies of the TriNetX network, data on the precise geographical location of patients were not available. Nevertheless, more than 95% of patients received care in hospitals located in the United States.

### Patient characteristics

Table 1 presents patient characteristics before and after propensity score matching. Before matching, 12 of the 44 characteristics differed between groups (StDiffs ≥0.10). Propensity score matching resulted in two comparable groups for baseline characteristics (all StDiffs <0.10). The majority of patients were male (64.5% of the overall population). The most prevalent comorbidity was diabetes, affecting 34.6% of patients. More than one-quarter of the overall population had a history of endocarditis. Notably, over 20% of the overall population had heart failure, cerebrovascular diseases, chronic lower respiratory diseases, or chronic kidney diseases, or were overweight or obese. Regarding indwelling devices, 20.1% of the overall population had cardiovascular implants, while orthopedic joint implants were reported in 5.9% of the cohort. Although baseline characteristics in the cefazolin and ASP groups appeared similar after matching, notable differences emerged during the follow-up period with respect to specific infectious foci and the prescription of additional antibiotics.

**TABLE 1:** Baseline characteristics of patients with methicillin-susceptible *Staphylococcus aureus* bacteremia in the cefazolin and antistaphylococcal penicillin groups before and after propensity score matching^*a*^

	Characteristics before the propensity score matching				Characteristics after the propensity score matching			
	Overall	Cefazolin	Antistaphylococcal penicillins	Std diff	Overall	Cefazolin	Antistaphylococcal penicillins	Std diff
	(*N* = 17,656)	(*N* = 15,687)	(*N* = 1,969)		(*N* = 3,908)	(*N* = 1,954)	(*N* = 1,954)	
		Mean ± SD	Mean ± SD			Mean ± SD	Mean ± SD	
		or	or			or	or	
	*n* (%)	*n* (%)	*n* (%)			*n* (%)	*n* (%)	
Demographic variables at index event date								
Age (years)		55.2 ± 21.4	51.4 ± 22.4	**0.171**		51.2 ± 22.5	51.5 ± 22.4	0.013
	
Male patients	11,367 64.4	10,083 64.3	1,284 65.2	0.020	2,519 64.5	1,246 63.8	1,273 65.1	0.029
Medical history before the index event date								
Acute myocardial infarction	2,496 14.1	2,251 14.3	245 12.4	0.056	461 11.8	219 11.2	242 12.4	0.036
Heart failure	5,238 30.2	4,824 30.8	504 25.6	**0.115**	981 25.1	479 24.5	502 25.7	0.027
Atherosclerosis	2,460 13.9	2,286 14.6	174 8.8	**0.179**	339 8.7	165 8.4	174 8.9	0.016
Cerebrovascular diseases	3,409 19.3	2,953 19.8	456 23.2	**0.107**	865 22.13	424 21.7	441 22.6	0.021
Other degenerative diseases of the nervous system	763 4.3	704 4.5	59 3.0	0.079	108 2.8	50 2.6	58 3.0	0.025
Chronic lower respiratory diseases	5,151	4,648	503	0.091	968	467	501	0.040
	29.2	29.6	25.5		24.8	23.9	25.6	
Systemic connective tissue disorders	766	691	75	0.030	140	66	74	0.022
	4.3	4.4	3.8		3.6	3.4	3.8	
Personal history of peptic ulcer disease	332	304	28	0.040	54	26	28	0.009
	1.9	1.9	1.4		1.4	1.3	1.4	
Diseases of the liver	3,929	3,524	405	0.046	775	374	401	0.035
	22.3	22.5	20.6		19.8	19.1	20.5	
Chronic viral hepatitis	1,131	972	159	0.073	293	138	155	0.033
	6.4	6.2	8.1		7.5	7.1	7.9	
Diabetes mellitus	7,342	6,667	675	**0.170**	1,354	680	674	0.006
	41.6	42.5	34.2		34.6	34.8	34.5	
Cerebral palsy and other paralytic syndromes	1,080	916	164	0.097	304	149	155	0.011
	6.1	5.8	8.3		7.8	7.6	7.9	
Chronic kidney disease	5,443	4,988	455	**0.196**	893	438	455	0.021
	30.8	31.8	23.1		22.9	22.4	23.3	
Dependence on renal dialysis	1,755	1,646	109	**0.183**	218	109	109	< 0.001
	9.9	10.5	5.5		5.6	5.6	5.6	
Malignant neoplasms of the lips, oral cavity, and pharynx	199	175	24	0.010	45	21	24	0.014
	1.1	1.1	1.2		1.2	1.1	1.2	
Malignant neoplasms of digestive organs	639	567	72	0.002	132	62	70	0.023
	3.6	3.6	3.7		3.4	3.2	3.6	
Malignant neoplasms of respiratory and intrathoracic organs	408	364	44	0.006	85	41	44	0.011
	2.3	2.3	2.2		2.2	2.1	2.3	
Malignant neoplasms of the bone and articular cartilage	92	82	10	0.002	20	10	10	< 0.001
	0.5	0.5	0.5		0.5	0.5	0.5	
Malignant neoplasms of mesothelial and soft tissue	140	126	14	0.011	29	15	14	0.006
	0.8	0.8	0.7		0.7	0.8	0.7	
Malignant neoplasms of the breast	431	401	30	0.073	62	32	30	0.008
	2.4	2.6	1.5		1.6	1.6	1.5	
Malignant neoplasms of female genital organs	211	199	12	0.068	25	13	12	0.006
	1.2	1.3	0.6		0.6	0.7	0.6	
Malignant neoplasms of male genital organs	413	380	33	0.053	64	31	33	0.008
	2.3	2.4	1.7		1.6	1.6	1.7	
Malignant neoplasms of the urinary tract	310	276	34	0.002	63	29	34	0.020
	1.8	1.8	1.7		1.6	1.5	1.7	
Malignant neoplasms of the eye, brain, and other parts of the central nervous system	97	84	13	0.016	28	15	13	0.012
	0.5	0.5	0.7		0.7	0.8	0.7	
Malignant neoplasms of the thyroid and other endocrine glands	80	69	11	0.017	21	10	11	0.007
	0.5	0.4	0.6		0.5	0.5	0.6	
Malignant neoplasms of ill-defined, other secondary, and unspecified sites	1,191	1,093	98	0.084	195	97	98	0.002
	6.7	7.0	5.0		5.0	5.0	5.0	
Malignant neuroendocrine tumor	67	57	10	0.022	20	10	10	<0.001
	0.4	0.4	0.5		0.5	0.5	0.5	
Malignant melanoma of the skin	130	120	10	0.032	21	11	10	0.007
	0.7	0.8	0.5		0.5	0.6	0.5	
Human immunodeficiency virus disease	212	189	23	0.003	47	24	23	0.005
	1.2	1.2	1.2		1.2	1.2	1.2	
Transplanted organ and tissue status	673	619	54	0.067	106	52	54	0.006
	3.8	3.9	2.7		2.7	2.7	2.8	
Overweight and obesity	5,771	5,331	440	**0.261**	902	462	440	0.027
	32.7	34.0	22.3		23.2	23.6	22.5	
Tobacco use	1,780	1,643	137	**0.125**	260	123	137	0.029
	10.1	10.5	7.0		6.7	6.3	7.0	
Alcohol dependence	1,256	1,125	131	0.020	251	121	130	0.019
	7.1	7.2	6.7		6.4	6.2	6.7	
Acute and subacute endocarditis	2,848	2,269	579	**0.367**	1,136	572	564	0.009
	16.1	14.5	29.4		29.1	29.3	28.9	
Non-rheumatic mitral valve disorder	2,722	2,437	285	0.030	559	278	281	0.004
	15.4	15.5	14.5		14.3	14.2	14.4	
Non-rheumatic aortic valve disorder	2,225	1,987	238	0.018	447	213	234	0.034
	12.6	12.7	12.1		11.4	10.9	12.0	
Non-rheumatic tricuspid valve disorder	1,865	1,688	177	0.059	350	173	177	0.007
	10.6	10.8	9.0		9.0	8.9	9.1	
Non-rheumatic pulmonary valve disorder	600	554	46	0.071	80	34	46	0.043
	3.4	3.5	2.3		2.0	1.7	2.4	
Endocarditis, valve unspecified	2,406	2,026	380	**0.174**	735	367	368	0.001
	13.6	12.9	19.3		18.8	18.8	18.8	
Chronic rheumatic heart diseases	3,036	2,686	350	0.017	643	300	343	0.059
	17.2%	17.1%	17.8%		16.5%	15.4%	17.6%	
Presence of cardiac and vascular implants and grafts	4,185	3,789	396	0.097	785	393	392	0.001
	23.7	24.2	20.1		20.1	20.1	20.1	
Presence of orthopedic joint implants	1,921	1,810	111	**0.212**	230	119	111	0.017
	10.9	11.5	5.6		5.9	6.1	5.7	

^*a*^ *n*, number of instances; SD, standard deviation; St diffs, standardized difference; %, percentage. Boldfaced values indicate statistically significant differences between groups.

Table 2 presents the specific infectious foci associated with bacteremia documented after the index event. Patients in the cefazolin group were more frequently diagnosed with catheter-related infections (4.4% vs 2.7%). In contrast, the ASP group had a higher prevalence of central nervous system infections (2.9% vs 5.4%), native endocarditis (10.3% vs 12.0%), and device-related cardiovascular infections (2.7% vs 4.6%).

**TABLE 2:** Specific infectious foci diagnosed during the follow-up by groups in patients with methicillin-susceptible *Staphylococcus aureus* bacteremia^*a*^^,^^*b*^

Specific infectious foci	Cefazolin	Antistaphylococcal penicillins
	(*N* = 1,954)	(*N* = 1,954)
	*n* (%)	*n* (%)
Meningitis, encephalitis, and central nervous system abscess	57 2.9	106 5.4
Native endocarditis	201 10.3	235 12.0
Infection of cardiac and/or vascular device	52 2.7	90 4.6
Vertebral osteomyelitis	71 3.6	94 4.8
Native arthritis and non-vertebral osteomyelitis	142 7.3	147 7.5
Device-related arthritis and non-vertebral osteomyelitis	21 1.1	18 0.9
Catheter-related infection	126 4.4	76 2.7
Pneumonia	37 1.9	40 2.0
Skin infectious diseases	103 5.3	121 6.2

^*a*^ *n*, number of instances; %, percentage.

^*b*^ Each patient could have had none, one, or more than one documented infectious focus associated with bacteremia.

Other antibiotic prescriptions following the index event are presented in Table 3. For each class of additional antibiotics studied, only co-prescription of other β-lactams and glycopeptides concerned more than 10% of patients in each group. Notably, glycopeptides were more commonly prescribed in the cefazolin group, whereas quinolones, aminoglycosides, and rifampicin were more frequently used in the ASP group.

**TABLE 3:** Other antibiotics prescribed during the follow-up by group for methicillin-susceptible *Staphylococcus aureus* bacteremia^*a*^^*,*^^*b*^

Specific infectious foci	Cefazolin	Antistaphylococcal penicillins
	(*N* = 1,954)	(*N* = 1,954)
	*n* (%)	*n* (%)
Other β-lactams (cefazolin and ASPs excluded)	193 9.9	206 10.5
Glycopeptides	294 15.0	241 12.3
Lipoglycopeptides	43 2.2	27 1.4
Daptomycin	94 4.8	83 4.2
Oxazolidinones	52 2.7	50 2.6
Quinolones	73 3.7	93 4.8
	
Trimethoprim and/or sulfamethoxazole	59	53
	3.0	2.7
Macrolides, lincosamides, and streptogramins	74	88
	3.8	4.0
Aminoglycosides	132	165
	6.8	8.4
Rifampicin	118	167
	6.0	8.5
Tetracyclines	47	53
	2.4	2.7
Imidazole antibiotics	96	66
	4.9	3.4

^*a*^ Each patient could have received none, one, or more than one of these additional antibiotics.

^*b*^ ASP, antistaphylococcal penicillin; *n*, number of instances; %, percentage.

### Effectiveness outcomes

Patients who died prior to the time windows (between days 0 and 1) were excluded from these analyses (48 patients in the cefazolin group and 71 patients in the ASPs group).

**Fig 2:**
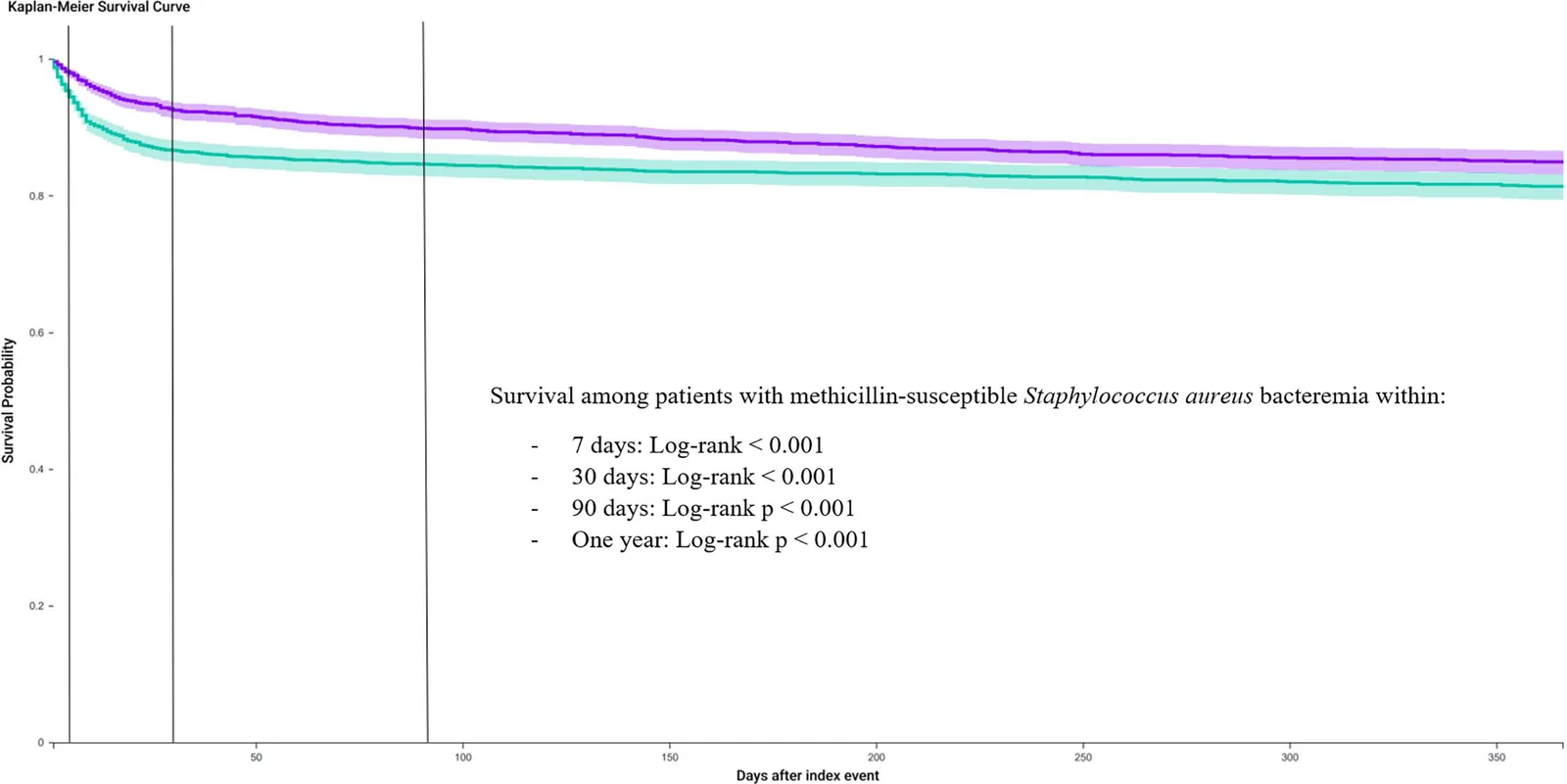
Comparison of survival among patients with methicillin-susceptible *Staphylococcus aureus* bacteremia between the propensity score-matched cefazolin and antistaphylococcal penicillin groups. The cefazolin group is represented in purple (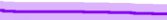). The antistaphylococcal penicillin group is represented in green (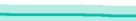). Patients who died prior to the time windows (between days 0 and 1) were excluded from these analyses (48 patients in the cefazolin group and 71 patients in the ASP group).

The survival curves for all-cause mortality in both propensity score-matched groups and the results of the log-rank tests are presented in Fig. 2. All-cause mortality within 90 days was significantly lower in the cefazolin group (log-rank *P* value <0.001). This difference remained significant across all time frames analyzed, including within 7 days, within 90 days, and up to 1 year (log-rank *P* value <0.001 in all cases).

Table 4 presents the risk and hazard ratios for these outcomes. The cefazolin group was associated with reduced all-cause mortality within 7 days (HR = 0.407 [0.299–0.553]), 30 days (HR = 0.535 [0.435–0.659]), 90 days (HR = 0.620 [0.515–0.748]), and 1 year (HR = 0.739 [0.627–0.871]).

**TABLE 4:** Comparison of all-cause mortality after propensity score matching in patients with methicillin-susceptible *Staphylococcus aureus* bacteremia between the cefazolin and antistaphylococcal penicillin groups

Effectiveness outcome	Patients with the outcome		Risk ratio (95% CI)	Follow-up time (days)		Hazard ratio (95% CI)	*P* value for hazard ratio
	*n* (%)			Mean ± SD			
	Cefazolin	Antistaphylococcal penicillins		Cefazolin	Antistaphylococcal penicillins		
	(*N* = 1,906)^*a*^	(*N* = 1,883)^*a*^		(*N* = 1,906)^*a*^	(*N* = 1,883)^*a*^		
All-cause mortality at 7 days	58	137	0.418	6.9 ± 0.8	6.7 ± 1.3	0.407	0.368
	3.0	7.3	(0.310–0.565)			(0.299–0.553)	
All-cause mortality at 30 days	139	243	0.565	28.1 ± 6.2	26.1 ± 8.7	0.535	0.001
	7.3	12.9	(0.463–0.689)			(0.435–0.659)	
All-cause mortality at 90 days	184	276	0.659	77.5 ± 26.3	71.6 ± 32.2	0.620	<0.001
	9.7	14.7	(0.553–0.785)			(0.515–0.748)	
All-cause mortality at one year	255	323	0.780	260.5 ± 140.8	250.1 ± 152.6	0.739	<0.001
	13.4	17.2	(0.670–0.907)			(0.627–0.871)	

^*a*^ Patients who died prior to the time windows (between days 0 and 1) were excluded from these analyses (48 patients in the cefazolin group and 71 patients in the ASP group).

### Sensitivity analysis

**Fig 3:**
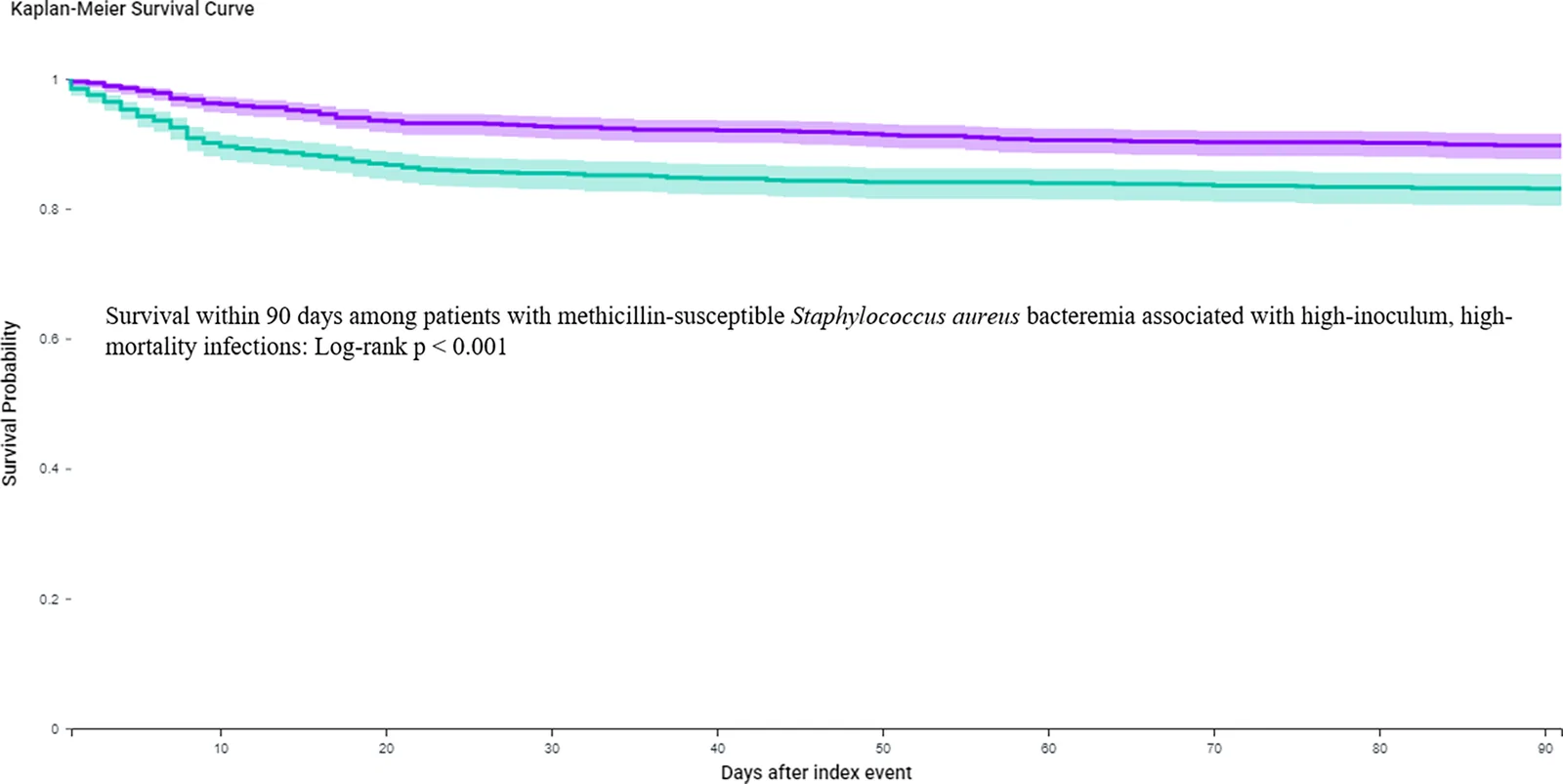
Comparison of survival among patients with methicillin-susceptible *Staphylococcus aureus* bacteremia associated with high-inoculum, high-mortality infections, between propensity score-matched cefazolin and antistaphylococcal penicillin groups (sensitivity analysis). The cefazolin group is represented in purple (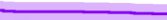). The antistaphylococcal penicillin group is represented in green (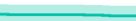). Patients who died prior to the time windows (between days 0 and 1) were excluded from these analyses (20 patients in the cefazolin group and 28 patients in the ASP group).

Before the propensity score matching, the subgroup of patients with methicillin-susceptible *Staphylococcus aureus* bacteremia associated with high-inoculum, high-mortality infections treated with cefazolin included 5,069 patients, while the ASP subgroup included 1,002 patients. After propensity score matching, each subgroup included 987 patients. For the analysis, 20 patients in the cefazolin subgroup and 28 patients in the ASP subgroup were excluded because they had the outcome prior to the time window (between days 0 and 1). All-cause mortality within 90 days was significantly lower in the cefazolin group (log-rank *P* value <0.001, HR = 0.563 [0.436–0.726]) (Fig. 3).

### Safety outcomes

**Fig 4:**
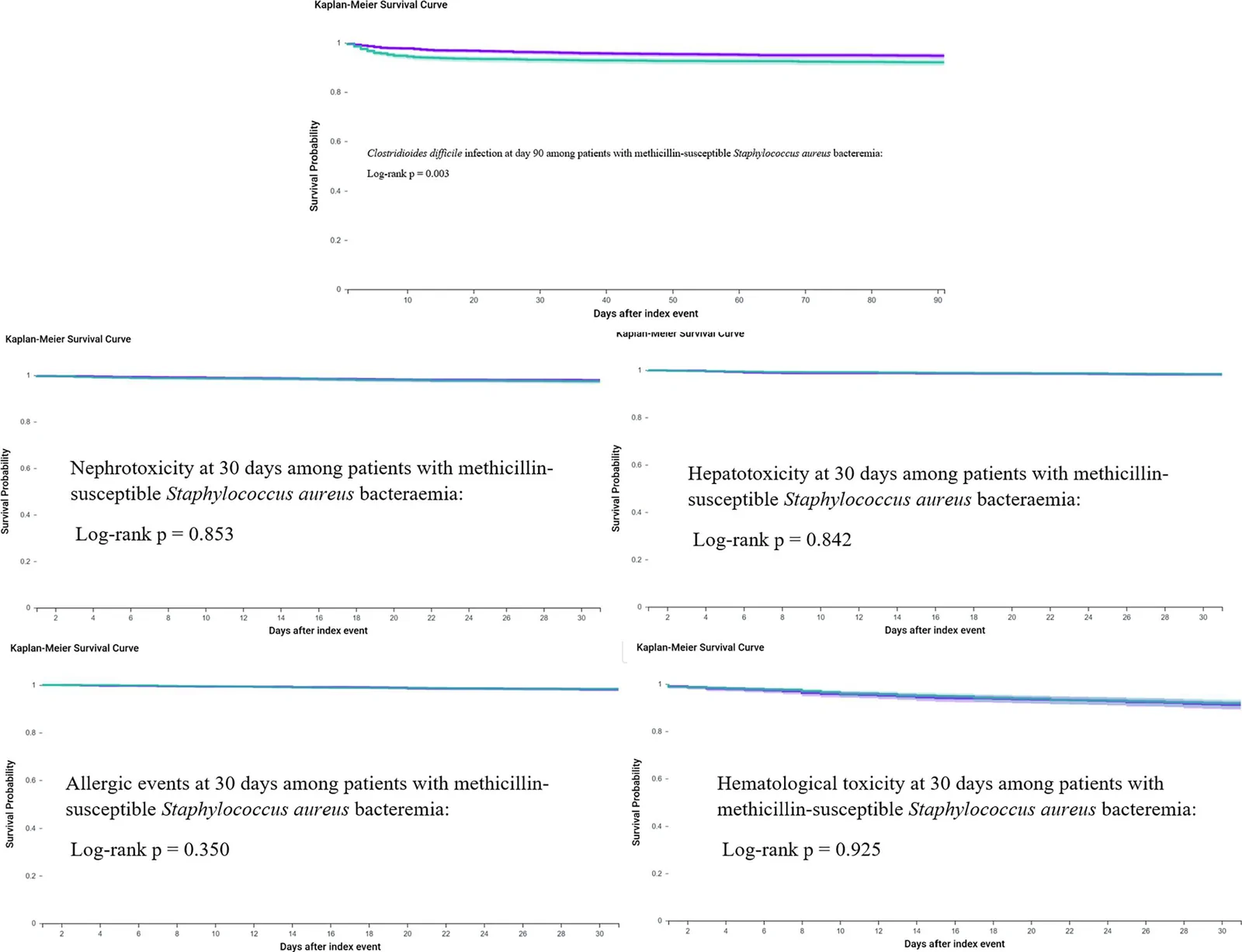
Comparison of safety outcomes among patients with methicillin-susceptible *Staphylococcus aureus* bacteremia between the propensity score-matched cefazolin and antistaphylococcal penicillin groups. The cefazolin group is represented in purple (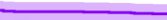). The antistaphylococcal penicillin group is represented in green (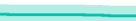). Patients who died prior to the time windows (between day 0 and 1) were excluded from these analyses (20 patients in the cefazolin group and 28 patients in the ASP group).

Secondary safety outcomes are presented in Fig. 4 and Table 5. Following propensity score matching, *C. difficile* infection at 90 days was observed more frequently in the ASP group. Conversely, no significant differences were observed between the two groups regarding renal impairment, liver dysfunction, allergic events, and hematological disorders at 30 days. Moreover, compared with the use of ASPs, cefazolin for the treatment of MSSA bacteremia appeared to be associated with a lower incidence of *C. difficile* infection at 90 days (log-rank *P* value of 0.001, HR = 0.631 [0.482–0.827]).

**TABLE 5:** Comparison of safety outcomes after propensity score matching in patients with methicillin-susceptible *Staphylococcus aureus* bacteremia between the cefazolin and antistaphylococcal penicillin groups^*a*^

Safety outcome	Patients with the outcome		Risk ratio (95% CI)	Follow-up time (days)		Hazard ratio (95% CI)	*P* value for hazard ratio
	*n* (%)			Mean ± SD			
	Cefazolin	Antistaphylococcal penicillins		Cefazolin	Antistaphylococcal penicillins		
*Clostridioides difficile* infection at 90 days	88/1,802	131/1,808	0.674	77.5 ± 26.3	71.0 ± 32.2	0.631	0.003
	4.90	7.20	(0.519–0.876)			(0.482–0.827)	
Nephrotoxicity at 30 days	25/1,217	28/1,173	0.861	28.1 ± 6.2	26.1 ± 8.7	0.831	0.853
	2.10	2.40	(0.505–1.467)			(0.485–1.425)	
Hepatotoxicity at 30 days	28/1,658	25/1,633	1.103	28.1 ± 6.2	26.1 ± 8.7	1.057	0.409
	1.50	1.5	(0.646–1.883)			(0.616–1.812)	
Allergic events at 30 days	29/1,680	25/1,736	1.199	28.1 ± 6.2	26.1 ± 8.7	1.122	0.350
	1.70	1.40	(0.705–2,038)			(0.657–1.915)	
Hematological toxicity at 30 days	83/990	69/936	1.137	28.1 ± 6.2	26.1 ± 8.7	1.086	0.925
	8.40	7.40	(0.837–1.545)			(0.789–1.494)	

^*a*^ Patients who experienced the outcome prior the time windows (before the index event or between days 0 and 1) were excluded from these analyses: *Clostridioides difficile* infection at day 90: 152 patients in the cefazolin group and 146 patients in the ASP group; nephrotoxicity at 30 days: 737 patients in the cefazolin group and 781 patients in the ASPs group; hepatotoxicity at 30 days: 296 patients in the cefazolin group and 321 patients in the ASP group; allergic events at 30 days: 274 patients in the cefazolin group and 218 patients in the ASP group; hematological toxicity at 30 days: 964 patients in the cefazolin group and 1,018 patients in the ASP group.

## DISCUSSION

In this observational study including 3,908 matched patients, cefazolin treatment was associated with lower all-cause mortality within 90 days among patients hospitalized with MSSA bacteremia. Moreover, the incidence of *C. difficile* infection within 90 days was significantly lower among patients receiving cefazolin compared with those treated with ASPs. Surprisingly, despite the existence of conflicting data in the literature, a majority of patients were treated with cefazolin in this study. One possible explanation is that most patients were managed in the United States. This observation is consistent with a recent practice survey by Westgeest et al., which reported that the majority of North American clinicians use cefazolin as first-line therapy for MSSA bacteremia (10). Nevertheless, several other explanations may account for these findings: (i) previous observational studies may have influenced clinical practices, prompting clinicians to favor cefazolin when managing patients with MSSA bacteremia (7, 9–11); (ii) the cost of cefazolin is lower than that of ASPs (48, 49); and (iii) the greater stability of cefazolin in solution facilitates administration (50–54).

### Comparison with literature

The findings of this study are consistent with the majority of the recent literature comparing cefazolin with ASPs in MSSA bacteremia. Indeed, since 2018, eight meta-analyses have compiled data from observational studies on this topic (34, 36–42) (Table S1). Most concluded that cefazolin is likely non-inferior, or potentially superior, to ASPs in terms of all-cause mortality while also presenting a more favorable safety profile. Nevertheless, the authors of these meta-analyses consistently highlight methodological heterogeneity and inherent biases in the included observational studies. These limitations hinder the ability to draw definitive clinical conclusions. Recently, the results of the CloCeBa RCT and the findings of the *Staphylococcus aureus* Network Adaptive Platform (SNAP) were published (43, 44). They corroborate findings from previous observational studies (Table S1). The findings of our study further strengthen this growing body of evidence.

Among the literature, seven meta-analyses compared all-cause mortality (36–42) (Table S1). Cefazolin was consistently associated with improved effectiveness in terms of mortality outcomes across various time points. However, in the CloCeBa trial (43), the all-cause mortality was evaluated at 90 days and was identical in both groups (12/146, i.e., 8.2%). In 2026, the 90-day all-cause mortality did not differ between groups in the SNAP trial (97/645, i.e., 15.0% in the cefazolin group and 109/642, i.e., 17.0% in the ASP group) (44). Notably, the magnitude of the mortality difference between treatment groups appears smaller in recent RCTs than in meta-analyses of observational studies. Several factors may account for this discrepancy, including better control of confounding in randomized trials, as well as methodological aspects, such as non-inferiority trial designs, more selected patient populations, and stricter inclusion criteria.

With respect to safety, eight meta-analyses and the two RCTs compared cefazolin with ASPs (34, 36–44). The incidence of *C. difficile* infection was explored only in the two RCTs, which did not find any difference between groups (43, 44). Our study is the first to report a lower incidence of this infection in the cefazolin group. It is important to note that the classical definitions of this outcome used in international guidelines could not be directly applied within the TriNetX database (55). Nevertheless, substantial effort was made to construct an operational definition that closely approximates international standards. Importantly, the same definition was applied to both treatment groups; therefore, although some degree of misclassification may have led to a slight overestimation of the outcome in absolute terms, there is no reason to believe that this misclassification differed between groups. Most previous studies have reported a lower risk of renal impairment associated with cefazolin compared with ASPs (34, 36, 39, 42–44); however, this increased risk of renal complications associated with ASPs was not observed in our findings. The most likely explanation relates to the definition of renal impairment used in our analysis. Renal impairment was identified solely based on clinician-recorded diagnoses of acute kidney injury and corresponding healthcare organization coding. Because the TriNetX database does not provide standardized longitudinal laboratory measurements of renal function for all patients, it was not possible to apply the KDIGO criteria for acute kidney injury. Consequently, milder or unrecognized cases of acute kidney injury may not have been captured, leading to an underestimation of the true incidence of renal impairment. Liver dysfunction is a classical adverse event reported in the literature. Most studies suggested better hepatic safety with cefazolin, although this difference was not statistically significant in our study (34, 36). Consistent with our findings, one meta-analysis and the two RCTs reported no significant difference in allergic reaction rates (36, 43, 44), and similar results were found regarding hematological disorders (34, 43, 44). Finally, some studies instead used “treatment discontinuation due to toxicity” or “adverse drug reaction” as surrogate markers for toxicity (34, 36–38, 40, 41, 43, 44), often reporting fewer events with cefazolin; unfortunately, such variables were not available in the TriNetX database for our study.

Among the published meta-analyses, only a few reported the specific infectious foci associated with MSSA bacteremia (36–41), and none described the additional antibiotic prescriptions received by patients. In our study, a lower mortality rate was observed among patients treated with cefazolin at the different time points. Nevertheless, the group treated with ASPs included a higher proportion of high-inoculum infections (e.g., endocarditis and meningitis), which are known to be associated with a poorer prognosis (6). A similar pattern was observed among patients who died on the index event day, with mortality rates of 2.4% (48/1,969) in the cefazolin group and 3.6% (71/1,969) in the ASP-treated group. This finding further supports the likelihood that patients receiving ASPs had a poorer baseline prognosis, probably reflecting a greater proportion of high-inoculum infections. As in the meta-analysis (36–41), part of the observed mortality difference in our study may therefore be attributable to the higher frequency of high-inoculum infections in the ASP group, and then the mortality differences we observed between the two groups were probably slightly overestimated.

Moreover, our study provided data on other antibiotic prescriptions, offering complementary insight into the complete antibiotic treatment received by patients. These additional antibiotics were unlikely to have been intended for the treatment of the bacteremia itself but rather for empirical therapy or for the management of other MSSA infection sites following initial intravenous treatment with cefazolin or ASPs (e.g., oral treatment of spondylodiscitis and arthritis). As in the CloCeBa trial (43), only a few patients received antibiotic co-prescriptions, and the differences between the two groups were minor. In the SNAP trial, pre-enrollment antibiotic co-prescriptions did not differ, and post-enrollment administration of additional antibiotics was discouraged (44). Therefore, it is reasonable to consider that the observed differences in effectiveness and safety outcomes were more likely attributable to the use of cefazolin vs ASPs rather than to the influence of these antibiotic co-prescriptions.

Compared with the CloCeBa and SNAP trials (43, 44), there are numerous differences that make any comparison of results challenging. These differences concern the number of patients included (3,908 patients vs 292 and 1,341 patients, respectively), the study design (observational vs RCT), the characteristics of the enrolled patients related to recruitment methods (population-based study vs patients selected from RCT), the associated site of infection (a higher proportion of high-inoculum MSSA infections in our study), and the outcome measures (survival at various time points for our study and SNAP trial vs a composite endpoint including survival at day 90 for the CloCeBa trial).

### Strengths and limitations

The principal strengths of our study include its large sample size (3,908 patients), multicenter scope, and use of a large real-world global database, reflecting current practices. Although patients were not selected through the rigorous enrollment procedures typically used in randomized controlled trials, our inclusion and exclusion criteria were deliberately designed to identify a homogeneous study population. Indeed, the majority of patients with MSSA bacteremia recorded in the database were excluded. While this approach reduced the representativeness of the overall population of patients with MSSA bacteremia, it allowed the exclusion of patients with concomitant infections, recurrent episodes of MSSA bacteremia, or exposure to both antistaphylococcal β-lactam therapies during the index episode or within the preceding 3 months. This selection process was considered essential to ensure the inclusion of patients with a first, isolated episode of MSSA bacteremia. Without these restrictions, the study population would have been substantially more heterogeneous, thereby limiting the interpretability of the study outcomes.

Moreover, several limitations should be acknowledged. First, the observational design involves an inherent risk of confounding bias, which prevents causal inferences from being drawn. To limit this bias, propensity score matching was performed, including a large number of potential confounding factors, to improve initial comparability between cohorts. However, residual confounding may persist, particularly with respect to differences in infection foci associated with MSSA bacteremia and the severity of infection. Such residual imbalance may have contributed to an overestimation of the survival benefit observed in the cefazolin group. Second, although the TriNetX database is highly valuable because of its international scope and large patient population, it has several limitations. In particular, important variables such as detailed information on healthcare organizations, markers of infection severity, delay between infection diagnosis and treatment administration, or antibiotic regimens (e.g., dosing and duration) are unavailable. These missing pieces of information could have introduced biases into our analysis. Third, reliance on electronic health record data introduces a risk of misclassification, missing data, and residual confounding, despite rigorous variable definitions and statistical adjustments. Data within the TriNetX platform reflect routine documentation practices within participating healthcare organizations and cannot be assumed to be complete or entirely free from error, particularly with regard to safety outcomes such as renal or hepatic toxicity. Fourth, it is acknowledged that our study may have been subject to immortal time bias, as inclusion required survival until receipt of the treatment of interest. However, this potential bias is expected to be non-differential between groups, and there is no a priori reason to assume that it would have been more pronounced in one treatment group than in the other. In addition, patients may have received other antibiotics, or no antibiotic therapy, prior to initiation of an antistaphylococcal penicillin or cefazolin. Here again, there is no reason to expect that this pattern differed between the two treatment groups. Finally, the study was initially designed to leverage the international scope of the TriNetX network and to include the largest possible cohort of patients across multiple countries. However, the stringent eligibility criteria required to address the study hypothesis resulted in the exclusion of most patients with MSSA bacteremia, in order to obtain a clinically homogeneous population. Moreover, following additional information provided by TriNetX during the review process regarding the geographical distribution of patient care, it became apparent that the study population was predominantly composed of patients treated in the United States. Consequently, caution is warranted when extrapolating these findings to other healthcare systems. Nevertheless, our results are consistent with those of previous studies conducted in similar settings, supporting their external validity, particularly in high-income countries.

### Conclusion

This large observational study strengthens the existing evidence supporting the effectiveness and safety of cefazolin over ASPs for the treatment of MSSA bacteremia. Confirming the literature data, these findings invite a reconsideration of long-standing clinical assumptions regarding the optimal choice between these agents in the management of MSSA bacteremia.

## Data Availability

Data are available upon reasonable request. Researchers can request access to data from the TriNetX research network through the TriNetX platform (https://live.trinetx.com). However, this may involve associated costs and require a data-sharing agreement, and no patient-identifiable information can be accessible.

## SUPPLEMENTAL MATERIAL

Supplemental tables aac.01765-25-s0001.docx Tables S1 to S9.

ASM does not own the copyrights to Supplemental Material that may be linked to, or accessed through, an article. The authors have granted ASM a non-exclusive, world-wide license to publish the Supplemental Material files. Please contact the corresponding author directly for reuse.
