# Supplementary material for: Effectiveness and safety of cefazolin vs antistaphylococcal penicillins for methicillin-susceptible Staphylococcus aureus bacteremia: a multicenter real-world evaluation using the international TriNetX database
Source: Antimicrob Agents Chemother. 2026 Sep 11;70(10):e01765-25. doi: 10.1128/aac.01765-25 (PMC13688797; doi:10.1128/aac.01765-25)
Supplement: Supplementary file 1 [file aac.01765-25-s0001.docx]

**Supplementary Table 1:** Literature review on the effectiveness and safety of cefazolin versus antistaphylococcal penicillins in the treatment of methicillin-susceptible *Staphylococcus aureus* bacteraemia.

|  | Cefazolin group | Antistaphylococcal penicillins group | p-value |
| --- | --- | --- | --- |
|  |  |  |  |
| **All-cause mortality (%)** | | | |
| Eljaaly et al. 2018 | _ | _ | _ |
| Shi et al. 2018, defined at the study level | 375/1,782 (21.0%) | 711/2,997 (23.7%) | 0.033 |
| Rindone et al. 2018, defined at the study level | 295/1,774 (16.6%) | 712/2,954 (24.1%) | < 0.001 |
| Bidell et al. 2018, at 90 day | 289/1,589 (18.2%) | 703/2,802 (25.1%) | < 0.001 |
| Weiss et al. 2019, at 90 day | 289/1,589 (18.2%) | 703/2,802 (25.1%) | < 0.001 |
| Allen et al. 2019, defined at the study level | 394/3,081 (12.8%) | 1,483/10,309 (14.4%) | 0.025 |
| Lee et al. 2019, defined at the study level | 271/1,726 (15.7%) | 628/2,716 (23.1%) | < 0.001 |
| Prosty et al. 2025, at 90 day | 359/2,097 (17.1%) | 801/3,318 (24.1%) | < 0.001 |
| Burdet et al. 2025*, at day 90 | 12/146 (8.2%) | 12/146 (8.2%) | 1.000 |
| *Staphylococcus aureus* Network Adaptive Platform (SNAP) Trial Group 2026*, at day 90 | 97/645 (15.0%) | 109/642 (17.0%) | 0,343 |
| ***Clostridioides difficile* infection (%)** | | | |
| Eljaaly et al. 2018 | _ | _ | _ |
| Shi et al. 2018 | _ | _ | _ |
| Rindone et al. 2018 | _ | _ | _ |
| Bidell et al. 2018 | _ | _ | _ |
| Weiss et al. 2019 | _ | _ | _ |
| Allen et al. 2019 | _ | _ | _ |
| Lee et al. 2019 | _ | _ | _ |
| Prosty et al. 2025 | _ | _ | _ |
| Burdet et al. 2025*, until 7 days after the treatment discontinuation | 3/146 (2.1%) | 3/146 (2.1%) | 1.000 |
| *Staphylococcus aureus* Network Adaptive Platform (SNAP) Trial Group 2026*, at day 90 | 12/664 (2.1%) | 10/661 (1.5%) | 0,675 |
| **Nephrotoxicity (%)** | | | |
| Eljaaly et al. 2018, over the study course | 126/847 (14.9%) | 225/852 (26.4%) | < 0.001 |
| Shi et al. 2018 | _ | _ | _ |
| Rindone et al. 2018 | _ | _ | _ |
| Bidell et al. 2018 | _ | _ | _ |
| Weiss et al. 2019, over the study course | 17/499 (3.4%) | 82/689 (11.9%) | < 0.001 |
| Allen et al. 2019 | _ | _ | _ |
| Lee et al. 2019 | _ | _ | _ |
| Prosty et al. 2025, over the study course | 48/1,103 (4.4%) | 205/1,498 (13.7%) | < 0.001 |
| Burdet et al. 2025*, over the study course | 4/146 (2.7%) | 14/146 (9.6%) | 0.015 |
| *Staphylococcus aureus* Network Adaptive Platform (SNAP) Trial Group 2026*, at day 14 | 92/660 (13.9%) | 127/648 (19.6%) | 0,006 |
| **Hepatotoxicity (%)** | | | |
| Eljaaly et al. 2018, over the study course | 2/258 (0.8%) | 20/263 (7.6%) | < 0.001 |
| Shi et al. 2018 | _ | _ | _ |
| Rindone et al. 2018 | _ | _ | _ |
| Bidell et al. 2018 | _ | _ | _ |
| Weiss et al. 2019 | _ | _ | _ |
| Allen et al. 2019 | _ | _ | _ |
| Lee et al. 2019 | _ | _ | _ |
| Prosty et al. 2025 | _ | _ | _ |
| Burdet et al. 2025*, over the study course | 4/146 (2.7%) | 3/146 (2.1%) | 0.702 |
| *Staphylococcus aureus* Network Adaptive Platform (SNAP) Trial Group 2026*, at day 14 | 75/574 (13.1%) | 78/562 (13.9%) | 0.688 |
| **Allergic events (%)** | | | |
| Eljaaly et al. 2018 | _ | _ | _ |
| Shi et al. 2018 | _ | _ | _ |
| Rindone et al. 2018 | _ | _ | _ |
| Bidell et al. 2018 | _ | _ | _ |
| Weiss et al. 2019 | _ | _ | _ |
| Allen et al. 2019 | _ | _ | _ |
| Lee et al. 2019 | _ | _ | _ |
| Prosty et al. 2025 | _ | _ | _ |
| Burdet et al. 2025*, over the study course | 1/146 (0.7%) | 2/146 (1.4%) | 0.562 |
| *Staphylococcus aureus* Network Adaptive Platform (SNAP) Trial Group 2026*, serious adverse event over the study course | 0/671 (0%) | 0/670 (0%) | 1.000 |
| **Haemotological disorders events (%)** | | | |
| Eljaaly et al. 2018 | _ | _ | _ |
| Shi et al. 2018 | _ | _ | _ |
| Rindone et al. 2018 | _ | _ | _ |
| Bidell et al. 2018 | _ | _ | _ |
| Weiss et al. 2019 | _ | _ | _ |
| Allen et al. 2019 | _ | _ | _ |
| Lee et al. 2019 | _ | _ | _ |
| Prosty et al. 2025 | _ | _ | _ |
| Burdet et al. 2025*, over the study course | 6/146 (4.1%) | 2/146 (1.4%) | 0.099 |
| *Staphylococcus aureus* Network Adaptive Platform (SNAP) Trial Group 2026*, serious adverse event over the study course | 0/671 (0%) | 0/670 (0%) | 1.000 |
| **Treatment-related adverse events (%)** | | | |
| Eljaaly et al. 2018 | _ | _ | _ |
| Shi et al. 2018 | _ | _ | _ |
| Rindone et al. 2018 | _ | _ | _ |
| Bidell et al. 2018 | _ | _ | _ |
| Weiss et al. 2019 | _ | _ | _ |
| Allen et al. 2019 | _ | _ | _ |
| Lee et al. 2019, over the study course | 26/339 (7.7%) | 79/349 (22.6%) | < 0.001 |
| Prosty et al. 2025, over the study course | 116/1,152 (10.1%) | 412/1,755 (23.5%) | < 0.001 |
| Burdet et al. 2025*, over the study course | 97/146 (66.4%) | 102/146 (69.9%) | 0.530 |
| *Staphylococcus aureus* Network Adaptive Platform (SNAP) Trial Group 2026*, serious adverse event over the study course | 12/671 (1.8%) | 33/670 (4.9%) | 0.001 |
| **Discontinuation due to adverse reaction (%)** | | | |
| Eljaaly et al. 2018, over the study course | 1/239 (0.4%) | 24/227 (10.6%) | < 0.001 |
| Shi et al. 2018 | _ | _ | _ |
| Rindone et al. 2018 | 12/247 (4.9%) | 73/319 (22.9%) | < 0.001 |
| Bidell et al. 2018 | 12/179 (6.7%) | 62/238 (26.1%) | < 0.001 |
| Weiss et al. 2019 | _ | _ | _ |
| Allen et al. 2019, over the study course | 57/558 (10.2%) | 265/857 (30.9%) | < 0.001 |
| Lee et al. 2019 | _ | _ | _ |
| Prosty et al. 2025, over the study course | 14/669 (2.1%) | 142/843 (16.8%) | < 0.001 |
| Burdet et al. 2025*, over the study course | 5/146 (3.4%) | 13/146 (8.9%) | 0.052 |
| *Staphylococcus aureus* Network Adaptive Platform (SNAP) Trial Group 2026*, over the study course | 11/671 (1.6%) | 61/670 (9.1%) | < 0.001 |
|  |  |  |  |

Footnote: The time frame for the all-cause mortality outcome varied across the individual meta-analyses. * Theses publications are randomised controlled trial. All of the other publications are meta-analyses of observational studies.

**Supplementary Table 2.** TriNetX variables used to define the inclusion and exclusion criteria for patient selection in the cefazolin group.

| **Group 1** | | | | | |
| --- | --- | --- | --- | --- | --- |
|  | **Group 1A Methicillin-susceptible *Staphylococcus aureus* bacteraemia** | | | | |
|  | **Logical link** | | **Variables** | | |
|  |  |  | **Type** | **Code** | **Name** |
|  | must have |  | diagnosis | UMLS:ICD10CM:B95.61 | Methicillin susceptible *Staphylococcus aureus* infection as the cause of diseases classified elsewhere |
|  |  | and any of | diagnosis | UMLS:ICD10CM:T80.211 | Bloodstream infection due to central venous catheter |
|  |  |  | diagnosis | UMLS:ICD10CM:R78.81 | Bacteraemia |
|  | date constraint | | The terms in this group occurred at any time | | |
|  | event relationship | | Inclusion if cefazolin treatment occurred within 2 days before or up to 3 days after any instance of methicillin-susceptible *Staphylococcus aureus* bacteraemia | | |
|  | **Group 1B Inclusion of cefazolin treatment** | | | | |
|  | **Logical link** | | **Variables** | | |
|  |  |  | **Type** | **Code** | **Name** |
|  | must have | any of | medication | NLM:RXNORM:2180 | cefazolin (Route: Injectable Product) |
|  |  |  | procedure | UMLS:HCPCS:J0690 | Injection, cefazolin sodium, 500 mg |
|  |  |  | procedure | UMLS:HCPCS:J0689 | Injection, cefazolin sodium (baxter), not therapeutically equivalent to j0690, 500 mg |
|  |  |  | procedure | UMLS:HCPCS:J0688 | Injection, cefazolin sodium (hikma), not therapeutically equivalent to j0690, 500 mg |
| **Group 2** | | | | | |
|  | **Group 2A Methicillin-susceptible *Staphylococcus aureus* bacteraemia** | | | | |
|  | **Logical link** | | **Variables** | | |
|  |  |  | **Type** | **Code** | **Name** |
|  | must have |  | diagnosis | UMLS:ICD10CM:B95.61 | Methicillin susceptible *Staphylococcus aureus* infection as the cause of diseases classified elsewhere |
|  |  | and any of | diagnosis | UMLS:ICD10CM:T80.211 | Bloodstream infection due to central venous catheter |
|  |  |  | diagnosis | UMLS:ICD10CM:R78.81 | Bacteraemia |
|  | date constraint | | The terms in this group occurred at any time | | |
|  | event relationship | | Exclusion if antistaphylococcal penicillins treatment occurred within 3 months before or up to 3 months after any instance of methicillin-susceptible *Staphylococcus aureus* bacteraemia | | |
|  | **Group 2B Exclusion of antistaphylococcal penicillins treatment** | | | | |
|  | **Logical link** | | **Variables** | | |
|  |  |  | **Type** | **Code** | **Name** |
|  | cannot have |  | procedure | UMLS:HCPCS:J2700 | Injection, oxacillin sodium, up to 250 mg |
|  |  | or | medication | NLM:RXNORM:2625 | cloxacillin (Route: Injectable Product) |
|  |  | or | medication | NLM:RXNORM:7773 | oxacillin (Route: Injectable Product) |
|  |  | or | medication | NLM:RXNORM:7233 | nafcillin (Route: Injectable Product) |
|  |  | or | medication | NLM:RXNORM:3356 | dicloxacillin (Route: Injectable Product) |
|  |  | or | medication | NLM:RXNORM:4448 | floxacillin (Route: Injectable Product) |
|  |  | or | procedure | UMLS:HCPCS:S0032 | Injection, nafcillin sodium, 2 grams |
| **Group 3** | | | | | |
|  | **Group 3A Methicillin-susceptible *Staphylococcus aureus* bacteraemia** | | | | |
|  | **Logical link** | | **Variables** | | |
|  |  |  | **Type** | **Code** | **Name** |
|  | must have |  | diagnosis | UMLS:ICD10CM:B95.61 | Methicillin susceptible *Staphylococcus aureus* infection as the cause of diseases classified elsewhere |
|  |  | and any of | diagnosis | UMLS:ICD10CM:T80.211 | Bloodstream infection due to central venous catheter |
|  |  |  | diagnosis | UMLS:ICD10CM:R78.81 | Bacteremia |
|  | date constraint | | The terms in this group occurred at any time | | |
|  | event relationship | | Exclusion of patients with infection due to other bacterial pathogens occurred within 3 months before or up to 3 months after any instance of methicillin-susceptible *Staphylococcus aureus* bacteraemia | | |
|  | **Group 3B Exclusion of patient with infection due to other bacterial pathogens** | | | | |
|  | **Logical link** | | **Variables** | | |
|  |  |  | **Type** | **Code** | **Name** |
|  | cannot have |  | diagnosis | UMLS:ICD10CM:B95.0 | Streptococcus, group A, as the cause of diseases classified elsewhere |
|  |  | or | diagnosis | UMLS:ICD9CM:041.0 | Streptococcus infection in conditions classified elsewhere and of unspecified site |
|  |  | or | diagnosis | UMLS:ICD10CM:A41.3 | Sepsis due to *Haemophilus influenzae* |
|  |  | or | diagnosis | UMLS:ICD10CM:A36 | Diphtheria |
|  |  | or | diagnosis | UMLS:ICD10CM:A37 | Whooping cough |
|  |  | or | diagnosis | UMLS:ICD10CM:A32 | Listeriosis |
|  |  | or | diagnosis | UMLS:ICD10CM:A40 | Streptococcal sepsis |
|  |  | or | diagnosis | UMLS:ICD10CM:A41.4 | Sepsis due to anaerobes |
|  |  | or | diagnosis | UMLS:ICD10CM:A43 | Nocardiosis |
|  |  | or | diagnosis | UMLS:ICD10CM:A39 | Meningococcal infection |
|  |  | or | diagnosis | UMLS:ICD10CM:A44 | Bartonellosis |
|  |  | or | diagnosis | UMLS:ICD10CM:A38 | Scarlet fever |
|  |  | or | diagnosis | UMLS:ICD10CM:A42 | Actinomycosis |
|  |  | or | diagnosis | UMLS:ICD10CM:A48 | Other bacterial diseases, not elsewhere classified |
|  |  | or | diagnosis | UMLS:ICD10CM:A41.5 | Sepsis due to other Gram-negative organisms |
|  |  | or | diagnosis | UMLS:ICD10CM:B96 | Other bacterial agents as the cause of diseases classified elsewhere |
|  |  | or | diagnosis | UMLS:ICD10CM:B95.5 | Unspecified streptococcus as the cause of diseases classified elsewhere |
|  |  | or | diagnosis | UMLS:ICD10CM:B95.4 | Other streptococcus as the cause of diseases classified elsewhere |
|  |  | or | diagnosis | UMLS:ICD10CM:A41.8 | Other specified sepsis |
|  |  | or | diagnosis | UMLS:ICD10CM:B95.3 | Streptococcus pneumoniae as the cause of diseases classified elsewhere |
|  |  | or | diagnosis | UMLS:ICD10CM:B95.2 | Enterococcus as the cause of diseases classified elsewhere |
|  |  | or | diagnosis | UMLS:ICD10CM:B95.1 | Streptococcus, group B, as the cause of diseases classified elsewhere |

Footnotes: To be included in the cefazolin group, patients must meet all inclusion and exclusion criteria outlined in groups 1, 2, and 3.

Abbreviations:

- CPT = Current Procedural Terminology

- ICD9CM: International Classification of Diseases, 9th Revision, Clinical Modification

- ICD10CM = International Classification of Diseases, 10th Revision, Clinical Modification

- HCPCS = Healthcare Common Procedure Coding System

- LNC= Logical Observation Identifiers Names and Codes

- NLM = National Library of Medicine

- RxNorm = normalized naming system for generic and branded drugs

- UMLS = Unified Medical Language System

**Supplementary Table 3.** TriNetX variables used to define the inclusion and exclusion criteria for patient selection in the antistaphylococcal penicillins group.

| **Group 1** | | | | | |
| --- | --- | --- | --- | --- | --- |
|  | **Group 1A Methicillin-susceptible *Staphylococcus aureus* bacteraemia** | | | | |
|  | **Logical link** | | **Variables** | | |
|  |  |  | **Type** | **Code** | **Name** |
|  | must have |  | diagnosis | UMLS:ICD10CM:B95.61 | Methicillin susceptible *Staphylococcus aureus* infection as the cause of diseases classified elsewhere |
|  |  | and any of | diagnosis | UMLS:ICD10CM:T80.211 | Bloodstream infection due to central venous catheter |
|  |  |  | diagnosis | UMLS:ICD10CM:R78.81 | Bacteraemia |
|  | date constraint | | The terms in this group occurred at any time | | |
|  | event relationship | | Inclusion if antistaphylococcal penicillins treatment occurred within 2 days before or up to 3 days after any instance of methicillin-susceptible *Staphylococcus aureus* bacteraemia | | |
|  | **Group 1B Inclusion of antistaphylococcal penicillins treatment** | | | | |
|  | **Logical link** | | **Variables** | | |
|  |  |  | **Type** | **Code** | **Name** |
|  | must have | any of | procedure | UMLS:HCPCS:J2700 | Injection, oxacillin sodium, up to 250 mg |
|  |  |  | procedure | UMLS:HCPCS:S0032 | Injection, nafcillin sodium, 2 grams |
|  |  |  | medication | NLM:RXNORM:3356 | dicloxacillin (Route: Injectable Product) |
|  |  |  | medication | NLM:RXNORM:4448 | floxacillin (Route: Injectable Product) |
|  |  |  | medication | NLM:RXNORM:2625 | cloxacillin (Route: Injectable Product) |
|  |  |  | medication | NLM:RXNORM:7773 | oxacillin (Route: Injectable Product) |
|  |  |  | medication | NLM:RXNORM:7233 | nafcillin (Route: Injectable Product) |
| **Group 2** | | | | | |
|  | **Group 2A Methicillin-susceptible *Staphylococcus aureus* bacteraemia** | | | | |
|  | **Logical link** | | **Variables** | | |
|  |  |  | **Type** | **Code** | **Name** |
|  | must have |  | diagnosis | UMLS:ICD10CM:B95.61 | Methicillin susceptible *Staphylococcus aureus* infection as the cause of diseases classified elsewhere |
|  |  | and any of | diagnosis | UMLS:ICD10CM:T80.211 | Bloodstream infection due to central venous catheter |
|  |  |  | diagnosis | UMLS:ICD10CM:R78.81 | Bacteraemia |
|  | date constraint | | The terms in this group occurred at any time | | |
|  | event relationship | | Exclusion if cefazolin treatment occurred within 3 months before or up to 3 months after any instance of methicillin-susceptible *Staphylococcus aureus* bacteraemia | | |
|  | **Group 2B Exclusion of cefazolin treatment** | | | | |
|  | **Logical link** | | **Variables** | | |
|  |  |  | **Type** | **Code** | **Name** |
|  | cannot have |  | medication | NLM:RXNORM:2180 | cefazolin (Route: Injectable Product) |
|  |  | or | procedure | UMLS:HCPCS:J0690 | Injection, cefazolin sodium, 500 mg |
|  |  | or | procedure | UMLS:HCPCS:J0689 | Injection, cefazolin sodium (baxter), not therapeutically equivalent to j0690, 500 mg |
|  |  | or | procedure | UMLS:HCPCS:J0688 | Injection, cefazolin sodium (hikma), not therapeutically equivalent to j0690, 500 mg |
| **Group 3** | | | | | |
|  | **Group 3A Methicillin-susceptible *Staphylococcus aureus* bacteraemia** | | | | |
|  | **Logical link** | | **Variables** | | |
|  |  |  | **Type** | **Code** | **Name** |
|  | must have |  | diagnosis | UMLS:ICD10CM:B95.61 | Methicillin susceptible *Staphylococcus aureus* infection as the cause of diseases classified elsewhere |
|  |  | and any of | diagnosis | UMLS:ICD10CM:T80.211 | Bloodstream infection due to central venous catheter |
|  |  |  | diagnosis | UMLS:ICD10CM:R78.81 | Bacteraemia |
|  | date constraint | | The terms in this group occurred at any time | | |
|  | event relationship | | Exclusion of patients with infection due to other bacterial pathogens occurred within 3 month before or up to 3 month after any instance of methicillin-susceptible *Staphylococcus aureus* bacteraemia | | |
|  | **Group 3B Exclusion of patients with infection due to other bacterial pathogens** | | | | |
|  | **Logical link** | | **Variables** | | |
|  |  |  | **Type** | **Code** | **Name** |
|  | cannot have |  | diagnosis | UMLS:ICD10CM:B95.0 | Streptococcus, group A, as the cause of diseases classified elsewhere |
|  |  | or | diagnosis | UMLS:ICD9CM:041.0 | Streptococcus infection in conditions classified elsewhere and of unspecified site |
|  |  | or | diagnosis | UMLS:ICD10CM:A41.3 | Sepsis due to *Haemophilus influenzae* |
|  |  | or | diagnosis | UMLS:ICD10CM:A36 | Diphtheria |
|  |  | or | diagnosis | UMLS:ICD10CM:A37 | Whooping cough |
|  |  | or | diagnosis | UMLS:ICD10CM:A32 | Listeriosis |
|  |  | or | diagnosis | UMLS:ICD10CM:A40 | Streptococcal sepsis |
|  |  | or | diagnosis | UMLS:ICD10CM:A41.4 | Sepsis due to anaerobes |
|  |  | or | diagnosis | UMLS:ICD10CM:A43 | Nocardiosis |
|  |  | or | diagnosis | UMLS:ICD10CM:A39 | Meningococcal infection |
|  |  | or | diagnosis | UMLS:ICD10CM:A44 | Bartonellosis |
|  |  | or | diagnosis | UMLS:ICD10CM:A38 | Scarlet fever |
|  |  | or | diagnosis | UMLS:ICD10CM:A42 | Actinomycosis |
|  |  | or | diagnosis | UMLS:ICD10CM:A48 | Other bacterial diseases, not elsewhere classified |
|  |  | or | diagnosis | UMLS:ICD10CM:A41.5 | Sepsis due to other Gram-negative organisms |
|  |  | or | diagnosis | UMLS:ICD10CM:B96 | Other bacterial agents as the cause of diseases classified elsewhere |
|  |  | or | diagnosis | UMLS:ICD10CM:B95.5 | Unspecified streptococcus as the cause of diseases classified elsewhere |
|  |  | or | diagnosis | UMLS:ICD10CM:B95.4 | Other streptococcus as the cause of diseases classified elsewhere |
|  |  | or | diagnosis | UMLS:ICD10CM:A41.8 | Other specified sepsis |
|  |  | or | diagnosis | UMLS:ICD10CM:B95.3 | Streptococcus pneumoniae as the cause of diseases classified elsewhere |
|  |  | or | diagnosis | UMLS:ICD10CM:B95.2 | Enterococcus as the cause of diseases classified elsewhere |
|  |  | or | diagnosis | UMLS:ICD10CM:B95.1 | Streptococcus, group B, as the cause of diseases classified elsewhere |

Footnotes: To be included in the antistaphylococcal penicillins group, patients must meet all inclusion and exclusion criteria outlined in groups 1, 2, and 3.

Abbreviations:

- CPT = Current Procedural Terminology

- ICD9CM: International Classification of Diseases, 9th Revision, Clinical Modification

- ICD10CM = International Classification of Diseases, 10th Revision, Clinical Modification

- HCPCS = Healthcare Common Procedure Coding System

- LNC= Logical Observation Identifiers Names and Codes

- NLM = National Library of Medicine

- RxNorm = normalized naming system for generic and branded drugs

- UMLS = Unified Medical Language System

**Supplementary Table 4.** TriNetX covariates used to define baseline patient characteristics.

| **Demographic** | | |  |
| --- | --- | --- | --- |
| Code in the TriNetX database | Name of the variable | Informations available about this variable |  |
| AI | Age at Index | Age at inclusion (corresponding to the date of the index event) |  |
|  |  |  |  |
| M | Male | Gender of the patient |  |
|  |  |  |  |
| **Diagnosis** | | |  |
| Code in the TriNetX database | Name of the variable | Informations available regarding this variable |  |
| I21 | Acute myocardial infarction | Patient's medical history of acute myocardial infarction (with or without ST elevation).  This includes myocardial infarction specified as acute or with a stated duration of 4 weeks (28 days) or less from onset, corresponding to the following ICD10CM codes: I21.0 acute transmural myocardial infarction of anterior wall; I21.1 acute transmural myocardial infarction of inferior wall; I21.2 Acute transmural myocardial infarction of other site; I21.3 acute transmural myocardial infarction of unspecified site, I21.4 acute subendocardial myocardial infarction, I21.5 acute myocardial infarction, unspecified, and I21.A other types of myocardial infarction. |  |
| I50 | Heart failure | Patient's medical history of heart failure (congestive, systolic, diastolic, combined…).  This includes congestive heart diseases, corresponding to the following ICD10CM codes: I50.1 left ventricular failure, I50.2 systolic (congestive) heart failure, I50.3 diastolic (congestive) heart failure, I50.4 combined systolic (congestive) and diastolic (congestive) heart failure, I50.8 other heart failure, I50.9 heart failure unspecificed |  |
| I70 | Atherosclerosis | Patient's medical history of atherosclerosis (aorta, its branches, native arteries, and the arteries of the extremities…).  This includes arteriolosclerosis, arteriosclerosis, arteriosclerotic vascular disease, atheroma degeneration, endarteritis deformans or obliterans and senile arteritis, corresponding to the following ICD10CM codes: I70.0 atherosclerosis of aorta, I70.1 atherosclerosis of renal artery, I70.2 atherosclerosis of native arteritis of the extremities, I70.3 atherosclerosis of unspecified type of bypass graft(s) ff the extremities, I70.4 atherosclerosis of autologous vein bypass graft(s) of the extremities, I70.5 atherosclerosis of nonautologous biological bypass graft(s) of the extremities, I70.6 atherosclerosis of nonbiological bypass graft(s) of the extremities, I70.7 atherosclerosis of other type of bypass graft(s) of the extremities, I70.8 atherosclerosis of other arteries, I70.9 other unspecified atherosclerosis. |  |
| I60-I69 | Cerebrovascular diseases | Patient's medical history of cerebrovascular diseases (cerebral infarction, occlusion/stenosis of cerebral arteries, cerebral atherosclerosis, cerebral aneurysm, hypertensive encephalopathy, non-pyogenic thrombosis, …).  This includes cerebrovascular diseases, corresponding to the following ICD10CM codes: I60 nontraumatic subarachnoid haemorrhage, I61 non traumatic intracerebral haemorrhage, I62 other an unspecified non traumatic intracranial haemorrhage, I63 cerebral infarction, I64 stroke, not specified as haemorrhage or infarction, I65 occlusion and stenosis of precerebral arteries, not resulting in cerebral infarction, I66 occlusion and stenosis of cerebral arteries, not resulting in cerebral infarction, I67 other cerebrovascular diseases, I68 cerebrovascular disorders in diseases classified elsewhere, I69 sequelae of cerebrovascular disease |  |
| G30-G32 | Other degenerative diseases of the nervous system | Patient's medical history of dementia (Alzheimer disease, fronto-temporal dementia, senile degeneration of the brain, …).  This includes central nervous system diseases, corresponding to the following ICD10CM codes: G30 Alzheimer disease, G31 other degenerative diseases of nervous system, not elsewhere classified (frontotemporal dementia, Pick disease, progressive isolated aphasia, senile degeneration of brain, degeneration of nervous system due to alcohol, Lewy body dementia, etc.), and G32 other degenerative disorders of nervous system in diseases classified elsewhere (Subacute combined degeneration of spinal cord and other specified degenerative disorders of nervous system in diseases classified elsewhere) |  |
| J40-J4A | Chronic lower respiratory diseases | Patient's medical history of chronic lower respiratory diseases (Chronic obstructive pulmonary diseases, asthma, emphysema, bronchiectasis, …).  This includes diseases of the respiratory system, corresponding to the following ICD10CM codes: J40  bronchitis, not specified as acute or chronic, J41  simple and mucopurulent chronic bronchitis, J42 unspecified chronic bronchitis, J43 emphysema, J44 other chronic obstructive pulmonary disease (Chronic obstructive pulmonary disease with acute lower respiratory infection or with acute exacerbation, etc), J45 asthma, J47 bronchiectasis, and J4A chronic lung allograft dysfunction. |  |
| M30-M36 | Systemic connective tissue disorders | Patient's medical history of systemic autoimmune diseases, such as systemic lupus erythematosus, necrotizing vasculopathies, systemic sclerosis, dermatomyositis/polymyositis, and polyarteritis nodosa…  This includes autoimmune diseases, corresponding to the following ICD10CM codes: M30 polyarteritis nodosa and related conditions, M31 other necrotizing vasculopathies, M32 systemic lupus erythematosus, M33 dermatopolymyositis, M34 systemic sclerosis, M35 other systemic involvement of connective tissue (Sjögren syndrome, overlap syndrome, Behçet disease, etc), and M36 Systemic disorders of connective tissue in diseases classified elsewhere (dermato(poly)myositis, and/or in neoplastic disease, etc) |  |
| Z87.11 | Personal history of peptic ulcer disease | Patient's medical history of gastric or duodenal peptic ulcer. |  |
| K70-K77 | Diseases of liver | Patient's medical history of liver disease whatever the etiologies, including steatosis, alcohol-related, toxic, or inflammatory causes, with or without cirrhosis.  This includes liver diseases, corresponding to the following ICD10CM codes: K70 alcoholic liver disease, K71 toxic liver disease (idiosyncratic/unpredictable or toxic/predictable liver diseases), K72 hepatic failure, not elsewhere classified, K73 chronic hepatitis, not elsewhere classified, K74 fibrosis and cirrhosis of liver, K75 other inflammatory liver diseases (abscess, phlebitis granulomatous, autoimmune, etc), K76  other diseases of liver (non-alcoholic fatty liver disease,  chronic passive congestion of liver, etc), and K77 liver disorders in diseases classified elsewhere (hepatic granulomas, infectious, and parasitic diseases). |  |
| B18 | Chronic viral hepatitis | Patient's medical history of chronic viral hepatitis, including each virus (B, C or Delta). |  |
| E08-E13 | Diabetes mellitus | Patient's medical history of diabetes diseases, including all etiologies (type 1, type 2, and secondary forms), both complicated and uncomplicated. |  |
| G80-G83 | Cerebral palsy and other paralytic syndromes | Patient's medical history of hemiplegia, paraplegia, quadriplegia, … |  |
| N18 | Chronic kidney disease (CKD) | Patient's medical history of chronic kidney disease, ranging from stage 2 to end-stage. |  |
| Z99.2 | Dependence on renal dialysis | Patient's medical history of dependence on renal dialysis due to chronic kidney disease. |  |
| C00-C14, C15-C26, C30-C39, C40-C41, C43,  C45-C49, C50-C50, C51-C58, C60-C63, C64-C68 C69-C72,  C73-C75, C7A-C7A &  C76-C80 | Malignant neoplasms | Patient's medical history of malignant neoplasm: lip, oral cavity, pharynx, digestive organs, lung, intrathoracic organs, bone, articular cartilage, mesothelium, soft tissue, breast, genital organs, urinary tract, central nervous system, endocrine glands, melanoma... |  |
| B20-B20 | Human immunodeficiency virus [HIV] disease (B20) | Patient's medical history of documented HIV infection. |  |
| Z94 | Transplanted organ and tissue status | Patient's medical history of solid organ or hematopoietic cell transplantation. |  |
| E66 | Overweight and obesity | Patient's medical history of body mass index superior or equal to 25. |  |
| Z72.0 | Tobacco use | Patient's medical history of tobacco use. |  |
| F10.2 | Alcohol dependence | Patient's medical history of dependence. |  |
| I33 & I38 | Acute and subacute endocarditis & Endocarditis, valve unspecified | Patient's medical history of infective endocarditis |  |
| I34 | Nonrheumatic mitral valve disorders | Patient's medical history of nonrheumatic valve diseases (insufficiency, prolapse, stenosis, …) |  |
| I35 | Nonrheumatic aortic valve disorders |  |  |
| I36 | Nonrheumatic tricuspid valve disorders |  |  |
| I37 | Nonrheumatic pulmonary valve disorders |  |  |
| I05-I09 | Chronic rheumatic heart diseases | Patient's medical history of rheumatic valve diseases (insufficiency, stenosis, …) |  |
| Z95 | Presence of cardiac and vascular implants and grafts | Patient's medical history of device implantation in cardiac and/or vascular tissues (valvular prosthesis, cardiac implantable electronic device, aorta endoprosthesis…) |  |
| Z96.6 | Presence of orthopaedic joint implants | Patient's medical history of device implantation in joints and/or bones (articular prosthesis, osteosynthesis, external fixator…) |  |

**Supplementary Table 5.** TriNetX covariate used to define the infectious foci related to the bacteraemia.

| **Definitions of bacteraemia related infections** | | | | |
| --- | --- | --- | --- | --- |
| **Logical link** | | **Variables** | | |
|  |  | **Type** | **Code** | **Name** |
| **Meningitis, encephalitis and central nervous system abscess** | | | | |
| must have |  | **Diagnosis** | UMLS:ICD10CM:G06 | Intracranial and intraspinal abscess and granuloma |
|  | or | **Diagnosis** | UMLS:ICD10CM:G00.3 | Staphylococcal meningitis |
|  | or | **Diagnosis** | UMLS:ICD9CM:320.7 | Meningitis in other bacterial diseases classified elsewhere |
|  | or | **Diagnosis** | UMLS:ICD10CM:G03.9 | Meningitis, unspecified |
|  | or | **Diagnosis** | UMLS:ICD10CM:G04.2 | Bacterial meningoencephalitis and meningomyelitis, not elsewhere classified |
|  | or | **Diagnosis** | UMLS:ICD9CM:320 | Bacterial meningitis |
|  | or | **Diagnosis** | UMLS:ICD10CM:G01 | Meningitis in bacterial diseases classified elsewhere |
| **Native endocarditis** | | | | |
| must have |  | **Diagnosis** | UMLS:ICD10CM:I38 | Endocarditis, valve unspecified |
|  | or | **Diagnosis** | UMLS:ICD9CM:421.1 | Acute and subacute infective endocarditis in diseases classified elsewhere |
|  | or | **Diagnosis** | UMLS:ICD9CM:424.91 | Endocarditis in diseases classified elsewhere |
|  | or | **Diagnosis** | UMLS:ICD10CM:I33 | Acute and subacute endocarditis |
| **Vertebral osteomyelitis** | | | | |
| must have |  | **Diagnosis** | UMLS:ICD10CM:M46.1 | Sacroiliitis, not elsewhere classified |
|  | or | **Diagnosis** | UMLS:ICD10CM:M46.2 | Osteomyelitis of vertebra |
|  | or | **Diagnosis** | UMLS:ICD10CM:M46.3 | Infection of intervertebral disc (pyogenic) |
|  | or | **Diagnosis** | UMLS:ICD10CM:M46.4 | Discitis, unspecified |
|  | or | **Diagnosis** | UMLS:ICD10CM:M46.5 | Other infective spondylopathies |
| **Native arthritis and non-vertebral osteomyelitis** | | | | |
| must have |  | **Diagnosis** | UMLS:ICD10CM:M00.0 | Staphylococcal arthritis and polyarthritis |
|  | or | **Diagnosis** | UMLS:ICD10CM:M00.9 | Pyogenic arthritis, unspecified |
|  | or | **Diagnosis** | UMLS:ICD10CM:M86 | Osteomyelitis |
| **Infection of cardiac and/or vascular device** | | | | |
| must have |  | **Diagnosis** | UMLS:ICD10CM:T82.6 | Infection and inflammatory reaction due to cardiac valve prosthesis |
|  | or | **Diagnosis** | UMLS:ICD10CM:T82.7 | Infection and inflammatory reaction due to other cardiac and vascular devices, implants and grafts |
| **Device related arthritis and non-vertebral osteomyelitis** | | | | |
|  | or | **Diagnosis** | UMLS:ICD10CM:T84.5 | Infection and inflammatory reaction due to internal joint prosthesis |
|  | or | **Diagnosis** | UMLS:ICD10CM:T84.61 | Infection and inflammatory reaction due to internal fixation device of arm |
|  | or | **Diagnosis** | UMLS:ICD10CM:T84.62 | Infection and inflammatory reaction due to internal fixation device of leg |
|  | or | **Diagnosis** | UMLS:ICD10CM:T84.7 | Infection and inflammatory reaction due to other internal orthopaedic prosthetic devices, implants and grafts |
| **Catheter related infection** | | | | |
| must have |  | **Diagnosis** | UMLS:ICD10CM:T80.21 | Infection due to central venous catheter |
| **Pneumonia** | | | | |
| must have |  | **Diagnosis** | UMLS:ICD10CM:J15.21 | Pneumonia due to *Staphylococcus aureus* |
|  | or | **Diagnosis** | UMLS:ICD10CM:J18 | Pneumonia, unspecified organism |
|  | or | **Diagnosis** | UMLS:ICD9CM:484.8 | Pneumonia in other infectious diseases classified elsewhere |
| **Skin infectious diseases** | | | | |
| must have |  | **Diagnosis** | UMLS:ICD10CM:A46 | Erysipelas |
|  | or | **Diagnosis** | UMLS:ICD10CM:L00-L08 | Infections of the skin and subcutaneous tissue |

Abbreviations:

- CPT = Current Procedural Terminology

- ICD9CM: International Classification of Diseases, 9th Revision, Clinical Modification

- ICD10CM = International Classification of Diseases, 10th Revision, Clinical Modification

- HCPCS = Healthcare Common Procedure Coding System

- LNC= Logical Observation Identifiers Names and Codes

- NLM = National Library of Medicine

- RxNorm = normalized naming system for generic and branded drugs

- UMLS = Unified Medical Language System

**Supplementary Table 6.** TriNetX covariates used to define the other antibiotics used.

| **Definition of other antibiotics used** | | | | |
| --- | --- | --- | --- | --- |
| **Logical link** | | **Variables** | | |
|  |  | **Type** | **Code** | **Name** |
| **Other Beta-lactam (excluding cefazolin and antistaphylococcal penicillins)** | | | | |
| must have |  | **Medication** | NLM:ATC:J01CA | Penicillins with extended spectrum |
|  | or | **Medication** | NLM:ATC:J01CE | Beta-lactamase sensitive penicillins |
|  | or | **Medication** | NLM:ATC:J01CG | Beta-lactamase inhibitors |
|  | or | **Medication** | NLM:ATC:J01DC | Second-generation cephalosporins |
|  | or | **Medication** | NLM:ATC:J01DD | Third-generation cephalosporins |
|  | or | **Medication** | NLM:ATC:J01DE | Fourth-generation cephalosporins |
|  | or | **Medication** | NLM:ATC:J01DF | Monobactams |
|  | or | **Medication** | NLM:ATC:J01DH | Carbapenems |
|  | or | **Medication** | NLM:ATC:J01DI | Other cephalosporins and penems |
| **Quinolones** | | | | |
| must have |  | **Medication** | NLM:ATC:J01M | QUINOLONE ANTIBACTERIALS |
| **Trimethoprim and/or sulfamethoxazole** | | | | |
| must have |  | **Medication** | NLM:RXNORM:10180 | sulfamethoxazole |
|  | or | **Medication** | NLM:RXNORM:10829 | trimethoprim |
| **Macrolides, lincosamides and streptogramins** | | | | |
| must have |  | **Medication** | NLM:ATC:J01F | MACROLIDES, LINCOSAMIDES AND STREPTOGRAMINS |
| **Aminoglycosides** | | | | |
| must have |  | **Medication** | NLM:ATC:J01G | AMINOGLYCOSIDE ANTIBACTERIALS |
| **Rifampicin** | | | | |
| must have |  | **Medication** | NLM:RXNORM:9384 | rifampin |
| **Tetracyclines** | | | | |
| must have |  | **Medication** | NLM:ATC:J01A | TETRACYCLINES |
| **Glycopeptides** | | | | |
| must have |  | **Medication** | NLM:RXNORM:11124 | vancomycin |
|  | or | **Medication** | NLM:RXNORM:57021 | teicoplanin |
| **Lipoglycopeptides** | | | | |
| must have |  | **Medication** | NLM:RXNORM:1539239 | dalbavancin |
|  | or | **Medication** | NLM:RXNORM:1547611 | oritavancin |
| **Oxazolidinones** | | | | |
| must have |  | **Medication** | NLM:RXNORM:1540825 | tedizolid |
|  | or | **Medication** | NLM:RXNORM:190376 | linezolid |
| **Daptomycin** | | | | |
| must have |  | **Medication** | NLM:RXNORM:22299 | daptomycin |
| **Imidazole antibiotics** | | | | |
| must have |  | **Medication** | NLM:RXNORM:36314 | secnidazole |
|  | or | **Medication** | NLM:RXNORM:6922 | metronidazole |

**Supplementary Table 7.** TriNetX variables used to define the subgroup of patients with high-inoculum, high-mortality infections treated with cefazolin.

| **Group 1** | | | | | |
| --- | --- | --- | --- | --- | --- |
|  | **Group 1A Methicillin-susceptible *Staphylococcus aureus* bacteraemia** | | | | |
|  | must have |  | diagnosis | UMLS:ICD10CM:B95.61 | Methicillin susceptible *Staphylococcus aureus* infection as the cause of diseases classified elsewhere |
|  |  | and any of | diagnosis | UMLS:ICD10CM:T80.211 | Bloodstream infection due to central venous catheter |
|  |  |  | diagnosis | UMLS:ICD10CM:R78.81 | Bacteremia |
|  | date constraint | | The terms in this group occurred between Jan 1, 2015 and Dec 31, 2025 | | |
|  | event relationship | | Any instance of high inoculum and mortality infections occurred within 7 days before or up to 7 days after the first instance of methicillin-susceptible *Staphylococcus aureus* bacteraemia | | |
|  | **Group 1B High inoculum and mortality infections** | | | | |
|  | must have | any of | diagnosis | UMLS:ICD9CM:320 | Bacterial meningitis |
|  |  |  | diagnosis | UMLS:ICD10CM:I33 | Acute and subacute endocarditis |
|  |  |  | diagnosis | UMLS:ICD9CM:320.7 | Meningitis in other bacterial diseases classified elsewhere |
|  |  |  | diagnosis | UMLS:ICD10CM:G03.9 | Meningitis, unspecified |
|  |  |  | diagnosis | UMLS:ICD10CM:G01 | Meningitis in bacterial diseases classified elsewhere |
|  |  |  | diagnosis | UMLS:ICD9CM:424.91 | Endocarditis in diseases classified elsewhere |
|  |  |  | diagnosis | UMLS:ICD10CM:G04.2 | Bacterial meningoencephalitis and meningomyelitis, not elsewhere classified |
|  |  |  | diagnosis | UMLS:ICD9CM:421.1 | Acute and subacute infective endocarditis in diseases classified elsewhere |
|  |  |  | diagnosis | UMLS:ICD10CM:T82.7 | Infection and inflammatory reaction due to other cardiac and vascular devices, implants and grafts |
|  |  |  | diagnosis | UMLS:ICD10CM:T85.73 | Infection and inflammatory reaction due to nervous system devices, implants and graft |
|  |  |  | diagnosis | UMLS:ICD10CM:G06 | Intracranial and intraspinal abscess and granuloma |
|  |  |  | diagnosis | UMLS:ICD10CM:T82.6 | Infection and inflammatory reaction due to cardiac valve prosthesis |
|  |  |  | diagnosis | UMLS:ICD10CM:I38 | Endocarditis, valve unspecified |
|  |  |  | diagnosis | UMLS:ICD10CM:G00.3 | Staphylococcal meningitis |
| **Group 2** | | | | | |
|  | **Group 2A Methicillin-susceptible *Staphylococcus aureus* bacteraemia** | | | | |
|  | must have |  | diagnosis | UMLS:ICD10CM:B95.61 | Methicillin susceptible Staphylococcus aureus infection as the cause of diseases classified elsewhere |
|  |  | and any of | diagnosis | UMLS:ICD10CM:T80.211 | Bloodstream infection due to central venous catheter |
|  |  |  | diagnosis | UMLS:ICD10CM:R78.81 | Bacteremia |
|  | date constraint | | The terms in this group occurred between Jan 1, 2015 and Dec 31, 2025 | | |
|  | event relationship | | Inclusion if cefazolin treatment occurred within 2 days before or up to 3 days after the first instance of methicillin-susceptible *Staphylococcus aureus* bacteraemia | | |
|  | **Group 2B Inclusion of cefazolin treatment** | | | | |
|  | must have | any of | procedure | UMLS:HCPCS:J0687 | Injection, cefazolin sodium (wg critical care), not therapeutically equivalent to j0690, 500 mg |
|  |  |  | medication | NLM:RXNORM:2180 | cefazolin (Route: Injectable Product) |
|  |  |  | procedure | UMLS:HCPCS:J0689 | Injection, cefazolin sodium (baxter), not therapeutically equivalent to j0690, 500 mg |
|  |  |  | procedure | UMLS:HCPCS:J0690 | Injection, cefazolin sodium, 500 mg |
|  |  |  | procedure | UMLS:HCPCS:J0688 | Injection, cefazolin sodium (hikma), not therapeutically equivalent to j0690, 500 mg |
| **Group 3** | | | | | |
|  | **Group 3A Methicillin-susceptible *Staphylococcus aureus* bacteraemia** | | | | |
|  | must have |  | diagnosis | UMLS:ICD10CM:B95.61 | Methicillin susceptible *Staphylococcus aureus* infection as the cause of diseases classified elsewhere |
|  |  | and any of | diagnosis | UMLS:ICD10CM:T80.211 | Bloodstream infection due to central venous catheter |
|  |  |  | diagnosis | UMLS:ICD10CM:R78.81 | Bacteremia |
|  | date constraint | | The terms in this group occurred between Jan 1, 2015 and Dec 31, 2025 | | |
|  | event relationship | | No instance of antistaphylococcal penicillins treatment occurred within 3 months before or up to 3 months after the first instance of Methicillin-susceptible *Staphylococcus aureus* bacteraemia | | |
|  | **Group 3B Exclusion of antistaphylococcal penicillins treatment** | | | | |
|  | cannot have |  | procedure | UMLS:HCPCS:S0032 | Injection, nafcillin sodium, 2 grams |
|  |  | or | medication | NLM:RXNORM:7773 | oxacillin (Route: Injectable Product) |
|  |  | or | procedure | UMLS:HCPCS:J2290 | Injection, nafcillin sodium, 20 mg |
|  |  | or | medication | NLM:RXNORM:4448 | floxacillin (Route: Injectable Product) |
|  |  | or | medication | NLM:RXNORM:3356 | dicloxacillin (Route: Injectable Product) |
|  |  | or | medication | NLM:RXNORM:7233 | nafcillin (Route: Injectable Product) |
|  |  | or | medication | NLM:RXNORM:2625 | cloxacillin (Route: Injectable Product) |
|  |  | or | procedure | UMLS:HCPCS:J2700 | Injection, oxacillin sodium, up to 250 mg |
| **Group 4** | | | | | |
|  | **Group 4A Methicillin-susceptible *Staphylococcus aureus* bacteraemia** | | | | |
|  | must have |  | diagnosis | UMLS:ICD10CM:B95.61 | Methicillin susceptible *Staphylococcus aureus* infection as the cause of diseases classified elsewhere |
|  |  | and any of | diagnosis | UMLS:ICD10CM:T80.211 | Bloodstream infection due to central venous catheter |
|  |  |  | diagnosis | UMLS:ICD10CM:R78.81 | Bacteremia |
|  | date constraint | | The terms in this group occurred between Jan 1, 2015 and Dec 31, 2025 | | |
|  | event relationship | | Exclusion of patient with infection due to other bacterial pathogens occurred within 3 months before or up to 3 months after the first instance of methicillin-susceptible *Staphylococcus aureus* bacteraemia | | |
|  | **Group 4B Exclusion of patient with infection due to other bacterial pathogens** | | | | |
|  | cannot have |  | diagnosis | UMLS:ICD10CM:B95.0 | Streptococcus, group A, as the cause of diseases classified elsewhere |
|  |  | or | diagnosis | UMLS:ICD9CM:041.0 | Streptococcus infection in conditions classified elsewhere and of unspecified site |
|  |  | or | diagnosis | UMLS:ICD10CM:A41.3 | Sepsis due to *Haemophilus influenzae* |
|  |  | or | diagnosis | UMLS:ICD10CM:A36 | Diphtheria |
|  |  | or | diagnosis | UMLS:ICD10CM:A37 | Whooping cough |
|  |  | or | diagnosis | UMLS:ICD10CM:A32 | Listeriosis |
|  |  | or | diagnosis | UMLS:ICD10CM:A40 | Streptococcal sepsis |
|  |  | or | diagnosis | UMLS:ICD10CM:A41.4 | Sepsis due to anaerobes |
|  |  | or | diagnosis | UMLS:ICD10CM:A43 | Nocardiosis |
|  |  | or | diagnosis | UMLS:ICD10CM:A39 | Meningococcal infection |
|  |  | or | diagnosis | UMLS:ICD10CM:A44 | Bartonellosis |
|  |  | or | diagnosis | UMLS:ICD10CM:A38 | Scarlet fever |
|  |  | or | diagnosis | UMLS:ICD10CM:A42 | Actinomycosis |
|  |  | or | diagnosis | UMLS:ICD10CM:A48 | Other bacterial diseases, not elsewhere classified |
|  |  | or | diagnosis | UMLS:ICD10CM:A41.5 | Sepsis due to other Gram-negative organisms |
|  |  | or | diagnosis | UMLS:ICD10CM:B96 | Other bacterial agents as the cause of diseases classified elsewhere |
|  |  | or | diagnosis | UMLS:ICD10CM:B95.5 | Unspecified streptococcus as the cause of diseases classified elsewhere |
|  |  | or | diagnosis | UMLS:ICD10CM:B95.4 | Other streptococcus as the cause of diseases classified elsewhere |
|  |  | or | diagnosis | UMLS:ICD10CM:A41.8 | Other specified sepsis |
|  |  | or | diagnosis | UMLS:ICD10CM:B95.3 | Streptococcus pneumoniae as the cause of diseases classified elsewhere |
|  |  | or | diagnosis | UMLS:ICD10CM:B95.2 | Enterococcus as the cause of diseases classified elsewhere |
|  |  | or | diagnosis | UMLS:ICD10CM:B95.1 | Streptococcus, group B, as the cause of diseases classified elsewhere |

Abbreviations:

- CPT = Current Procedural Terminology

- ICD9CM: International Classification of Diseases, 9th Revision, Clinical Modification

- ICD10CM = International Classification of Diseases, 10th Revision, Clinical Modification

- HCPCS = Healthcare Common Procedure Coding System

- LNC= Logical Observation Identifiers Names and Codes

- NLM = National Library of Medicine

- RxNorm = normalized naming system for generic and branded drugs

- UMLS = Unified Medical Language System

**Supplementary Table 8.** TriNetX variables used to define the subgroup of patients with high-inoculum, high-mortality infections treated with antistaphylococcal penicillins.

| **Group 1** | | | | | |
| --- | --- | --- | --- | --- | --- |
|  | **Group 1A Methicillin-susceptible *Staphylococcus aureus* bacteraemia** | | | | |
|  | must have |  | diagnosis | UMLS:ICD10CM:B95.61 | Methicillin susceptible *Staphylococcus aureus* infection as the cause of diseases classified elsewhere |
|  |  | and any of | diagnosis | UMLS:ICD10CM:T80.211 | Bloodstream infection due to central venous catheter |
|  |  |  | diagnosis | UMLS:ICD10CM:R78.81 | Bacteremia |
|  | date constraint | | The terms in this group occurred between Jan 1, 2015 and Dec 31, 2025 | | |
|  | event relationship | | Any instance of high inoculum and mortality infections occurred within 7 days before or up to 7 days after the first instance of Methicillin-susceptible *Staphylococcus aureus* bacteraemia | | |
|  | **Group 1B High inoculum and mortality infections** | | | | |
|  | must have | any of | diagnosis | UMLS:ICD9CM:320 | Bacterial meningitis |
|  |  |  | diagnosis | UMLS:ICD10CM:I33 | Acute and subacute endocarditis |
|  |  |  | diagnosis | UMLS:ICD9CM:320.7 | Meningitis in other bacterial diseases classified elsewhere |
|  |  |  | diagnosis | UMLS:ICD10CM:G03.9 | Meningitis, unspecified |
|  |  |  | diagnosis | UMLS:ICD10CM:G01 | Meningitis in bacterial diseases classified elsewhere |
|  |  |  | diagnosis | UMLS:ICD9CM:424.91 | Endocarditis in diseases classified elsewhere |
|  |  |  | diagnosis | UMLS:ICD10CM:G04.2 | Bacterial meningoencephalitis and meningomyelitis, not elsewhere classified |
|  |  |  | diagnosis | UMLS:ICD9CM:421.1 | Acute and subacute infective endocarditis in diseases classified elsewhere |
|  |  |  | diagnosis | UMLS:ICD10CM:T82.7 | Infection and inflammatory reaction due to other cardiac and vascular devices, implants and grafts |
|  |  |  | diagnosis | UMLS:ICD10CM:T85.73 | Infection and inflammatory reaction due to nervous system devices, implants and graft |
|  |  |  | diagnosis | UMLS:ICD10CM:G06 | Intracranial and intraspinal abscess and granuloma |
|  |  |  | diagnosis | UMLS:ICD10CM:T82.6 | Infection and inflammatory reaction due to cardiac valve prosthesis |
|  |  |  | diagnosis | UMLS:ICD10CM:I38 | Endocarditis, valve unspecified |
|  |  |  | diagnosis | UMLS:ICD10CM:G00.3 | Staphylococcal meningitis |
| **Group 2** | | | | | |
|  | **Group 2A Methicillin-susceptible *Staphylococcus aureus* bacteraemia** | | | | |
|  | must have |  | diagnosis | UMLS:ICD10CM:B95.61 | Methicillin susceptible *Staphylococcus aureus* infection as the cause of diseases classified elsewhere |
|  |  | and any of | diagnosis | UMLS:ICD10CM:T80.211 | Bloodstream infection due to central venous catheter |
|  |  |  | diagnosis | UMLS:ICD10CM:R78.81 | Bacteremia |
|  | date constraint | | The terms in this group occurred between Jan 1, 2015 and Dec 31, 2025 | | |
|  | event relationship | | Exclusion if cefazolin treatment occurred within 3 months before or up to 3 months after the first instance of Methicillin-susceptible *Staphylococcus aureus* bacteraemia | | |
|  | **Group 2B Exclusion of cefazolin treatment** | | | | |
|  | cannot have |  | procedure | UMLS:HCPCS:J0689 | Injection, cefazolin sodium (baxter), not therapeutically equivalent to j0690, 500 mg |
|  |  | or | procedure | UMLS:HCPCS:J0688 | Injection, cefazolin sodium (hikma), not therapeutically equivalent to j0690, 500 mg |
|  |  | or | procedure | UMLS:HCPCS:J0687 | Injection, cefazolin sodium (wg critical care), not therapeutically equivalent to j0690, 500 mg |
|  |  | or | medication | NLM:RXNORM:2180 | cefazolin (Route: Injectable Product) |
|  |  | or | procedure | UMLS:HCPCS:J0690 | Injection, cefazolin sodium, 500 mg |
| **Group 3** | | | | | |
|  | **Group 3A Methicillin-susceptible *Staphylococcus aureus* bacteraemia** | | | | |
|  | must have |  | diagnosis | UMLS:ICD10CM:B95.61 | Methicillin susceptible *Staphylococcus aureus* infection as the cause of diseases classified elsewhere |
|  |  | and any of | diagnosis | UMLS:ICD10CM:T80.211 | Bloodstream infection due to central venous catheter |
|  |  |  | diagnosis | UMLS:ICD10CM:R78.81 | Bacteremia |
|  | date constraint | | The terms in this group occurred between Jan 1, 2015 and Dec 31, 2025 | | |
|  | event relationship | | Inclusion if antistaphylococcal penicillins treatment occurred within 2 days before or up to 3 days after the first instance of Methicillin-susceptible *Staphylococcus aureus* bacteraemia | | |
|  | **Group 3B Inclusion of antistaphylococcal penicillins treatment** | | | | |
|  | must have | any of | procedure | UMLS:HCPCS:S0032 | Injection, nafcillin sodium, 2 grams |
|  |  |  | medication | NLM:RXNORM:7233 | nafcillin (Route: Injectable Product) |
|  |  |  | procedure | UMLS:HCPCS:J2700 | Injection, oxacillin sodium, up to 250 mg |
|  |  |  | medication | NLM:RXNORM:2625 | cloxacillin (Route: Injectable Product) |
|  |  |  | medication | NLM:RXNORM:3356 | dicloxacillin (Route: Injectable Product) |
|  |  |  | medication | NLM:RXNORM:7773 | oxacillin (Route: Injectable Product) |
|  |  |  | procedure | UMLS:HCPCS:J2290 | Injection, nafcillin sodium, 20 mg |
|  |  |  | medication | NLM:RXNORM:4448 | floxacillin (Route: Injectable Product) |
| **Group 4** | | | | | |
|  | **Group 4A Methicillin-susceptible *Staphylococcus aureus* bacteraemia** | | | | |
|  | must have |  | diagnosis | UMLS:ICD10CM:B95.61 | Methicillin susceptible *Staphylococcus aureus* infection as the cause of diseases classified elsewhere |
|  |  | and any of | diagnosis | UMLS:ICD10CM:T80.211 | Bloodstream infection due to central venous catheter |
|  |  |  | diagnosis | UMLS:ICD10CM:R78.81 | Bacteremia |
|  | date constraint | | The terms in this group occurred between Jan 1, 2015 and Dec 31, 2025 | | |
|  | event relationship | | Exclusion of patient with infection due to other bacterial pathogens occurred within 3 months before or up to 3 months after the first instance of Methicillin-susceptible *Staphylococcus aureus* bacteraemia | | |
|  | **Group 4B Exclusion of patient with infection due to other bacterial pathogens** | | | | |
|  | cannot have |  | diagnosis | UMLS:ICD10CM:B95.0 | Streptococcus, group A, as the cause of diseases classified elsewhere |
|  |  | or | diagnosis | UMLS:ICD9CM:041.0 | Streptococcus infection in conditions classified elsewhere and of unspecified site |
|  |  | or | diagnosis | UMLS:ICD10CM:A41.3 | Sepsis due to *Haemophilus influenzae* |
|  |  | or | diagnosis | UMLS:ICD10CM:A36 | Diphtheria |
|  |  | or | diagnosis | UMLS:ICD10CM:A37 | Whooping cough |
|  |  | or | diagnosis | UMLS:ICD10CM:A32 | Listeriosis |
|  |  | or | diagnosis | UMLS:ICD10CM:A40 | Streptococcal sepsis |
|  |  | or | diagnosis | UMLS:ICD10CM:A41.4 | Sepsis due to anaerobes |
|  |  | or | diagnosis | UMLS:ICD10CM:A43 | Nocardiosis |
|  |  | or | diagnosis | UMLS:ICD10CM:A39 | Meningococcal infection |
|  |  | or | diagnosis | UMLS:ICD10CM:A44 | Bartonellosis |
|  |  | or | diagnosis | UMLS:ICD10CM:A38 | Scarlet fever |
|  |  | or | diagnosis | UMLS:ICD10CM:A42 | Actinomycosis |
|  |  | or | diagnosis | UMLS:ICD10CM:A48 | Other bacterial diseases, not elsewhere classified |
|  |  | or | diagnosis | UMLS:ICD10CM:A41.5 | Sepsis due to other Gram-negative organisms |
|  |  | or | diagnosis | UMLS:ICD10CM:B96 | Other bacterial agents as the cause of diseases classified elsewhere |
|  |  | or | diagnosis | UMLS:ICD10CM:B95.5 | Unspecified streptococcus as the cause of diseases classified elsewhere |
|  |  | or | diagnosis | UMLS:ICD10CM:B95.4 | Other streptococcus as the cause of diseases classified elsewhere |
|  |  | or | diagnosis | UMLS:ICD10CM:A41.8 | Other specified sepsis |
|  |  | or | diagnosis | UMLS:ICD10CM:B95.3 | Streptococcus pneumoniae as the cause of diseases classified elsewhere |
|  |  | or | diagnosis | UMLS:ICD10CM:B95.2 | Enterococcus as the cause of diseases classified elsewhere |
|  |  | or | diagnosis | UMLS:ICD10CM:B95.1 | Streptococcus, group B, as the cause of diseases classified elsewhere |

Abbreviations:

- CPT = Current Procedural Terminology

- ICD9CM: International Classification of Diseases, 9th Revision, Clinical Modification

- ICD10CM = International Classification of Diseases, 10th Revision, Clinical Modification

- HCPCS = Healthcare Common Procedure Coding System

- LNC= Logical Observation Identifiers Names and Codes

- NLM = National Library of Medicine

- RxNorm = normalized naming system for generic and branded drugs

- UMLS = Unified Medical Language System

**Supplementary Table 9.** TriNetX variables used to define clinical outcomes.

| **Outcomes definition** | | | | |
| --- | --- | --- | --- | --- |
| **Logical link** | | **Variables** | | |
|  |  | **Type** | **Code** | **Name** |
| **Effectiveness outcomes** | | | | |
| **7, 30, 90-day and one year all-cause mortality** | | | | |
| must have |  | **Demographics** | Deceased | Deceased |
|  | or | **Diagnosis** | UMLS:ICD10CM:R99 | Ill-defined and unknown cause of mortality |
|  | or | **Diagnosis** | UMLS:ICD10CM:R99-R99 | Ill-defined and unknown cause of mortality (R99) |
| **Logical link** | | **Variables** | | |
|  |  | **Type** | **Code** | **Name** |
| **Safety outcomes** | | | | |
| **90-day *Clostridioides difficile* infection** | | | | |
| must have |  | **Diagnosis** | UMLS:ICD10CM:A04.7 | Enterocolitis due to *Clostridium difficile* |
|  | or | **Procedure** | UMLS:CPT:87493 | Infectious agent detection by nucleic acid (DNA or RNA); *Clostridium difficile*, toxin gene(s), amplified probe technique |
|  | or |  | UMLS:CPT:87803 | Infectious agent antigen detection by immunoassay with direct optical (ie, visual) observation; *Clostridium difficile* toxin A |
|  | or |  | UMLS:CPT:0107U | *Clostridium difficile* toxin(s) antigen detection by immunoassay technique, stool, qualitative, multiple-step method |
|  | or | **Laboratory** | UMLS:LNC:54067-4 | *Clostridioides difficile* toxin genes [Presence] in Stool by NAA with probe detection |
|  | or |  | UMLS:LNC:34713-8 | *Clostridioides difficile* toxin A+B [Presence] in Stool |
|  | or |  | UMLS:LNC:79177-2 | *Clostridioides difficile* glutamate dehydrogenase and toxins A+B [Presence] in Stool by Rapid immunoassay |
|  | or |  | UMLS:LNC:74822-8 | *Clostridioides difficile* toxin B tcdB gene [Presence] in Stool by NAA with probe detection |
|  | or |  | UMLS:LNC:82197-5 | *Clostridioides difficile* toxin A+B tcdA+tcdB genes [Presence] in Stool by NAA with non-probe detection |
|  | or |  | UMLS:LNC:80685-1 | *Clostridioides difficile* toxin A+B tcdA+tcdB genes [Presence] in Stool by NAA with probe detection |
|  | or |  | UMLS:LNC:10895-1 | *Clostridioides difficile* toxin B [Presence] in Stool |
|  | or |  | UMLS:LNC:46131-9 | *Clostridioides difficile* toxin B [Presence] in Stool by Cytotoxin tissue culture assay |
|  | or |  | UMLS:LNC:13957-6 | *Clostridioides difficile* toxin A [Presence] in Stool by Immunoassay |
|  | or |  | UMLS:LNC:6366-9 | *Clostridioides difficile* toxin B [Units/volume] in Specimen by Immunoassay |
|  |  |  |  |  |
|  | or |  | UMLS:LNC:101557-7 | *Clostridioides difficile* DNA [Presence] in Stool by NAA with probe detection |
|  | or |  | UMLS:LNC:6359-4 | *Clostridioides difficile* toxin A [Units/volume] in Stool by Immunoassay |
|  | or |  | UMLS:LNC:6364-4 | *Clostridioides difficile* toxin A+B [Units/volume] in Specimen by Immunoassay ( (most recent occurrence)) |
|  | or |  | UMLS:LNC:34468-9 | *Clostridioides difficile* toxin A+B [Presence] in Stool by Immunoassay |
|  | or |  | UMLS:LNC:6363-6 | *Clostridioides difficile* toxin A+B [Units/volume] in Stool by Immunoassay (most recent occurrence)) |
|  | or |  | UMLS:LNC:6362-8 | *Clostridioides difficile* toxin A+B [Presence] in Stool by Cytotoxin tissue culture assay |
| **30-day renal impairment** | | | | |
| must have |  | **Diagnosis** | UMLS:ICD10CM:N17 | Acute kidney failure |
| **30-day liver dysfunction** | | | | |
| must have |  | **Diagnosis** | UMLS:ICD10CM:K72.0 | Acute and subacute hepatic failure |
|  | or | **Diagnosis** | UMLS:ICD10CM:K72.9 | Hepatic failure, unspecified |
|  | or | **Diagnosis** | UMLS:ICD10CM:K76.9 | Liver disease, unspecified |
|  | or | **Diagnosis** | UMLS:ICD9CM:573 | Other disorders of liver |
|  | or | **Diagnosis** | UMLS:ICD10CM:R74.0 | Nonspecific elevation of levels of transaminase and lactic acid dehydrogenase [LDH] |
|  | or | **Diagnosis** | UMLS:ICD10CM:K71.0 | Toxic liver disease with cholestasis |
|  | or | **Diagnosis** | UMLS:ICD10CM:K71.1 | Toxic liver disease with hepatic necrosis |
|  | or | **Diagnosis** | UMLS:ICD10CM:K71.2 | Toxic liver disease with acute hepatitis |
|  | or | **Diagnosis** | UMLS:ICD10CM:R17 | Unspecified jaundice |
|  | or | **Diagnosis** | UMLS:ICD10CM:K71.6 | Toxic liver disease with hepatitis, not elsewhere classified |
| **30-day allergic events** | | | | |
| must have |  | **Diagnosis** | UMLS:ICD10CM:L51 | Erythema multiform |
|  | or | **Diagnosis** | UMLS:ICD10CM:L50.0 | Allergic urticaria |
|  | or | **Diagnosis** | UMLS:ICD10CM:L50.1 | Idiopathic urticaria |
|  | or | **Diagnosis** | UMLS:ICD10CM:L50.8 | Other urticaria |
|  | or | **Diagnosis** | UMLS:ICD10CM:L50.9 | Urticaria, unspecified |
|  | or | **Diagnosis** | UMLS:ICD10CM:L53.0 | Toxic erythema |
|  | or | **Diagnosis** | UMLS:ICD10CM:D72.12 | Drug rash with eosinophilia and systemic symptoms syndrome |
|  | or | **Diagnosis** | UMLS:ICD10CM:T78.4 | Other and unspecified allergy |
|  | or | **Diagnosis** | UMLS:ICD10CM:T78.2 | Anaphylactic shock, unspecified |
|  | or | **Diagnosis** | UMLS:ICD10CM:T88.6 | Anaphylactic reaction due to adverse effect of correct drug or medicament properly administered |
|  | or | **Diagnosis** | UMLS:ICD10CM:R21 | Rash and other nonspecific skin eruption |
| **30-day haematological disorders** | | | | |
| must have |  | **Diagnosis** | UMLS:ICD10CM:D61.811 | Other drug-induced pancytopenia |
|  | or | **Diagnosis** | UMLS:ICD10CM:D61.818 | Other pancytopenia |
|  | or | **Diagnosis** | UMLS:ICD10CM:D64.9 | Anaemia, unspecified |
|  | or | **Diagnosis** | UMLS:ICD10CM:D69.6 | Thrombocytopenia, unspecified |
|  | or | **Diagnosis** | UMLS:ICD10CM:D69.59 | Other secondary thrombocytopenia |
|  | or | **Diagnosis** | UMLS:ICD10CM:D70.2 | Other drug-induced agranulocytosis |
|  | or | **Diagnosis** | UMLS:ICD10CM:D70.8 | Other neutropenia |
|  | or | **Diagnosis** | UMLS:ICD10CM:D70.9 | Neutropenia, unspecified |
|  | or | **Diagnosis** | UMLS:ICD9CM:288.0 | Neutropenia |
|  | or | **Diagnosis** | UMLS:ICD10CM:D72.10 | Eosinophilia, unspecified |
|  | or | **Diagnosis** | UMLS:ICD10CM:D72.19 | Other eosinophilia |
|  | or | **Diagnosis** | UMLS:ICD9CM:288.3 | Eosinophilia |

Abbreviations:

- CPT = Current Procedural Terminology

- ICD9CM: International Classification of Diseases, 9th Revision, Clinical Modification

- ICD10CM = International Classification of Diseases, 10th Revision, Clinical Modification

- HCPCS = Healthcare Common Procedure Coding System

- LNC= Logical Observation Identifiers Names and Codes

- NLM = National Library of Medicine

- RxNorm = normalized naming system for generic and branded drugs

- UMLS = Unified Medical Language System
